# Identification of Human IKK-2 Inhibitors of Natural Origin (Part I): Modeling of the IKK-2 Kinase Domain, Virtual Screening and Activity Assays

**DOI:** 10.1371/journal.pone.0016903

**Published:** 2011-02-24

**Authors:** Esther Sala, Laura Guasch, Justyna Iwaszkiewicz, Miquel Mulero, Maria-Josepa Salvadó, Montserrat Pinent, Vincent Zoete, Aurélien Grosdidier, Santiago Garcia-Vallvé, Olivier Michielin, Gerard Pujadas

**Affiliations:** 1 Grup de Recerca en Nutrigenòmica, Departament de Bioquímica i Biotecnologia, Universitat Rovira i Virgili, Campus de Sescelades, Tarragona, Catalonia, Spain; 2 Centre Tecnològic de Nutrició i Salut, Reus, Catalonia, Spain; 3 Molecular Modeling Group, Swiss Institute of Bioinformatics, Quartier UNIL-Sorge, Lausanne, Switzerland; University of South Florida College of Medicine, United States of America

## Abstract

**Background:**

Their large scaffold diversity and properties, such as structural complexity and drug similarity, form the basis of claims that natural products are ideal starting points for drug design and development. Consequently, there has been great interest in determining whether such molecules show biological activity toward protein targets of pharmacological relevance. One target of particular interest is hIKK-2, a serine-threonine protein kinase belonging to the IKK complex that is the primary component responsible for activating NF-κB in response to various inflammatory stimuli. Indeed, this has led to the development of synthetic ATP-competitive inhibitors for hIKK-2. Therefore, the main goals of this study were **(a)** to use virtual screening to identify potential hIKK-2 inhibitors of natural origin that compete with ATP and **(b)** to evaluate the reliability of our virtual-screening protocol by experimentally testing the *in vitro* activity of selected natural-product hits.

**Methodology/Principal Findings:**

We thus predicted that 1,061 out of the 89,425 natural products present in the studied database would inhibit hIKK-2 with good ADMET properties. Notably, when these 1,061 molecules were merged with the 98 synthetic hIKK-2 inhibitors used in this study and the resulting set was classified into ten clusters according to chemical similarity, there were three clusters that contained only natural products. Five molecules from these three clusters (for which no anti-inflammatory activity has been previously described) were then selected for *in vitro* activity testing, in which three out of the five molecules were shown to inhibit hIKK-2.

**Conclusions/Significance:**

We demonstrated that our virtual-screening protocol was successful in identifying lead compounds for developing new inhibitors for hIKK-2, a target of great interest in medicinal chemistry. Additionally, all the tools developed during the current study (i.e., the homology model for the hIKK-2 kinase domain and the pharmacophore) will be made available to interested readers upon request.

## Introduction

Natural products (NPs) are a valuable source of inspiration as lead compounds for the design and development of new drug candidates [1]. In fact, over 60% of the current anticancer drugs are natural-product-related molecules (*i.e.*, either NPs or derivatives/analogues that have been inspired by a natural compound) [2]. This trend is a direct consequence of the large scaffold diversity of these molecules [2]–[4], which, together with properties such as structural complexity and drug similarity, support the claim that they are ideal starting points for medicinal chemistry [2], [5]. Despite these clear advantages, it is estimated that only 5 to 15% of the approximately 250,000 described higher plant species have been tested for some type of biological activity [4] (and the use of other organisms such as micro-organisms, insects, fungi or marine species for this aim is just beginning) [6], [7]. It has recently been suggested that bioinformatics/chemoinformatics tools could be used to screen large NP databases and so identify new bioactive molecules for specific targets [1], [4], [8]. One target of interest for NPs is the human inhibitor nuclear-factor κB kinase 2 (hIKK-2). This target is a serine-threonine protein kinase belonging to the IKK complex and is the primary component responsible for activating the nuclear-factor κB transcription factor (NF-κB) in response to inflammatory stimuli. Indeed, the importance of the NF-κB pathway in regulating the expression of the genes controlling cellular immune and inflammatory responses has motivated research groups in both academia and the pharmaceutical industry to devote increasing efforts toward developing synthetic ATP-competitive inhibitors for hIKK-2 [9]–[36], which could be of therapeutic use, e.g., in patients affected by chronic inflammatory diseases [37].

The hIKK-2 subunit is a polypeptide of 756 residues with a kinase domain in its 15–300 sequence segment. At present, there are only two entries in the Protein Data Bank (http://www.pdb.org; PDB) [38] for hIKK-2 (*i.e.*, PDB codes 3BRT and 3BRV), and in both cases, they correspond to the NEMO-binding region located at the C-terminal end of the protein [39] (therefore, no experimental structure for the kinase domain of hIKK-2 is known). Moreover, obtaining a homology model for this kinase domain is not trivial because the more similar protein in the PDB (*i.e.*, the catalytic domain of human ZIP kinase; PDB code 1YRP) has a pairwise sequence alignment with hIKK-2 of only 217 residues long, with a low percentage of sequence identity (*i.e.*, 32%). Despite this limitation, the great pharmacological interest of this target has led scientists to develop either ligand-based approaches [14], [40]–[42] or homology models for this domain [13]–[16], [19], [27], [35], [42]–[44] and to use them in virtual-screening (VS) or drug-design projects. Unfortunately, these pharmacophore hypotheses or homology models are not in the public domain.

Therefore, the main goals of this study were to **(a)** use VS to identify potential hIKK-2 inhibitors of natural origin that can compete with ATP and **(b)** evaluate the reliability of our VS protocol by experimentally testing the *in vitro* activity of selected NP hits. To achieve these goals, we **(1)** developed a homology model for the hIKK-2 kinase domain which could stand the test of our validation criteria, **(2)** docked ATP-competitive molecules known to be potent and specific inhibitors of hIKK-2 with this model [10], [11], [13], [15], [16], [18], [20]–[31], **(3)** identified which of the resulting poses were *knowledge-based coherent* by analyzing whether they satisfied the experimentally known generic binding features of ATP-competitive inhibitors of kinases [45], **(4)** used the knowledge-based coherent poses to derive a structure-based common pharmacophore containing the key intermolecular interactions between hIKK-2 and its inhibitors, **(5)** obtained exclusion volumes from our homology model and added them to the pharmacophore, **(6)** validated the selectivity of the resulting pharmacophore and of the VS process using a large database of kinase decoys [46] and ATP-competitive inhibitors for hIKK-2 that were not used during the pharmacophore building [47], **(7)** used the previously validated structure-based pharmacophore and VS protocol to find ATP-competitive inhibitors for hIKK-2 in a database of NPs [48], and, finally, **(8)** proved the reliability of the prediction by testing the inhibitory effect of some selected hits on hIKK-2 *in vitro*. In addition, all the tools developed during the current study (*i.e.*, the homology model for the hIKK-2 kinase domain and the pharmacophore) are available to interested readers upon request.

## Results and Discussion

### Homology-model description

**Figure 1:**
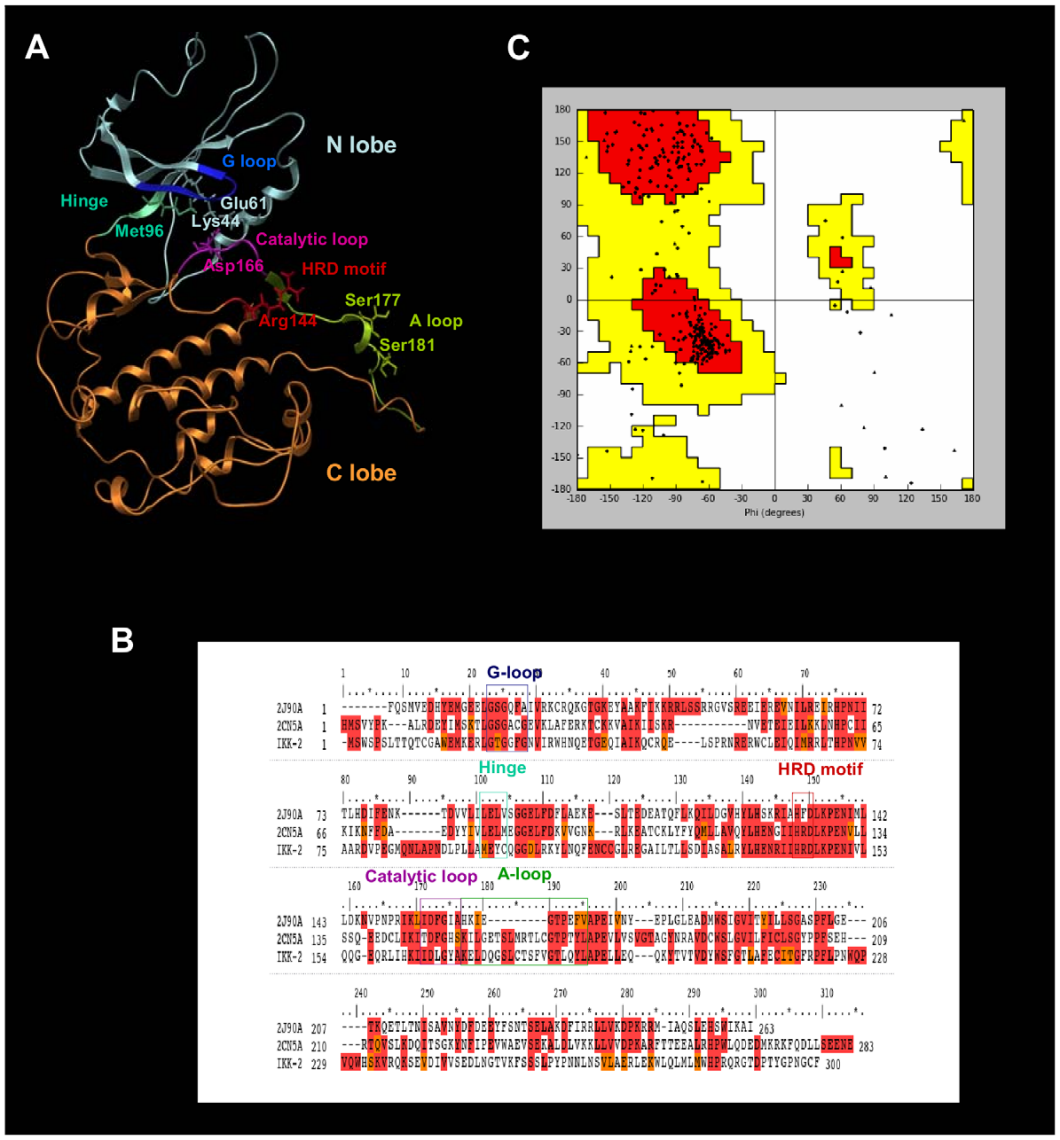
The homology model for the the kinase domain of hIKK-2. The best homology model for the kinase domain of hIKK-2 obtained in our study (Figure 1A), the multiple sequence alignment used to guide its modeling (Figure 1B) and its Ramachandran plot (Figure 1C). The relative locations of the G-loop, hinge region, HRD motif, catalytic loop and A-loop are shown in the model and in the sequence multialignment (using boxes) with the same color schema (*i.e.*, G-loop in blue; hinge region in cyan; HRD motif in red; catalytic loop in magenta and A-loop in green). The residues in the alignment are colored according to residue similarity. Circles in the Ramachandran plot show the location of non-glycine residues.

The protein kinase domains have a common fold consisting of two lobes linked by a flexible hinge region (see Figure 1A). Thus, the N-terminal lobe (from Trp15 to Ala95 in hIKK-2) is formed by a β-sheet (consisting of five antiparallel β-strands) and an α-helix that contains Glu61, which is one of the most important residues when hIKK-2 is in the active form because it makes polar contacts with the catalytic residue Lys44 (located in β3) [49], [50]. The N lobe also contains the highly conserved GXGXXG motif called the glycine-rich loop (G-loop) or P-loop, corresponding to the Gly22-Thr23-Gly24-Gly25-Phe26-Gly27 sequence segment in hIKK-2.

The C-terminal lobe (from Gln100 to Phe300 in hIKK-2) is highly helical and contains the activation segment formed by the HRD motif and the activation and catalytic loops. The HRD motif (from His143 to Asp145 in hIKK-2) is highly conserved in the kinases, and its arginine residue acts as the catalytic base that drives the phosphorylation event [50], [51]. The activation loop (A-loop; from Lys171 to Leu189 in hIKK-2) is the most flexible and diverse part of the activation segment [50] and includes residue(s) that when phosphorylated enable activity in many kinases (Ser177 and Ser181 for hIKK-2) [49], [52]. Finally, the catalytic loop (from Ile165 to Ala170 in hIKK-2) contains the DFG motif (but Asp166, Leu167 and Gly168 for hIKK-2) in most kinases, and its Asp residue forms polar contacts with all three ATP phosphates (either directly or through coordinating magnesium atoms).

The ATP binding site is a narrow, hydrophobic pocket located between the two lobes; its floor is formed by a C-terminal β-sheet, and its roof is formed by the G-loop, which fastens the γ-phosphate of ATP and serves as a regulatory flap above the ATP-binding site [45], [51], [53]–[55].

The most important residue in the hinge region of the protein kinase domain (from Met96 to Cys99 in hIKK-2) is the gatekeeper (*i.e.*, Met96 in hIKK-2) [18], which is located behind the hydrophobic pocket of the ATP-binding site. In that location, the size or volume of the side chain dictates its access to this pocket and therefore defines the potential inhibitor selectivity of the ATP-binding site. Moreover, this hinge region usually has a hydrogen donor flanked by hydrogen acceptors derived from the protein backbone, where they form a characteristic hydrogen-bond motif (in fact, the hinge region provides the most important hydrogen bonds with the ATP-site inhibitors). The residues involved in this hydrogen-bond motif are Glu97 and Cys99 in hIKK-2 (called gk+1 and gk+3 considering their relative location to the gatekeeper residue, *i.e.*, gk, in the hIKK-2 sequence) [18].

Figure 1B shows the multiple sequence alignment used to guide the modeling of the best 3D structure obtained for the hIKK-2 kinase domain, which is shown in Figure 1A. Because we were interested in finding new ATP-competitive inhibitors for hIKK-2, we selected two kinase structures as templates (2CN5 [56] and 2J90 [57]), each cocrystallized with a different ATP-competitive inhibitor (ADP and pyridone 6, respectively), both sharing a 32% sequence identity with the kinase domain of hIKK-2 and a 50% identity with the residues of the ATP-binding site of hIKK-2 (where these residues correspond to those in the G-loop, hinge region, HRD motif and catalytic loop).

The Ramachandran plot of this hIKK-2 homology model (see Figure 1C) shows that only 2.5% of the non-glycine residues (*i.e.*, Asn89, Gln110, Cys114, Arg144, Leu160, Thr185, Leu189 and Ala190) are in disallowed regions. Remarkably, none of these residues are located in the ATP-binding-site area and, therefore, its incorrect conformation did not affect our protein-ligand docking results. Moreover, when the ANOLEA results for the hIKK-2 model are compared with those for the two templates (see Table 1), the results show that the packing quality of our homology model is comparable to the packing qualities of the templates. Thus, although ANOLEA reports that 8 out of the 22 hIKK-2 high-energy residues (*i.e.*, from Phe182 to Leu189) are located in the A-loop (a very important area for kinase activity), it also shows that the equivalent residues in the two template structures also have high energies. The rest of high-energy residues in the hIKK-2 homology model are located in areas that are not crucial for its activity or structural stability.

**Table 1:** ANOLEA results for the two templates (2CN5 and 2J90) and the hIKK-2 homology model.

	2CN5A	2J90A	hIKK-2
**Total amino acids with high energy (percentage)**	22 (7.77)	31 (11.79)	22 (7.33)
**Total number of non-local atomic interactions**	32310	30226	33992
**Total non-local energy of the protein (E/kT units)**	−1878	−1946	−1956
**Non-local normalized energy Z-score**	0.04	−0.49	0.09

**Figure 2:**
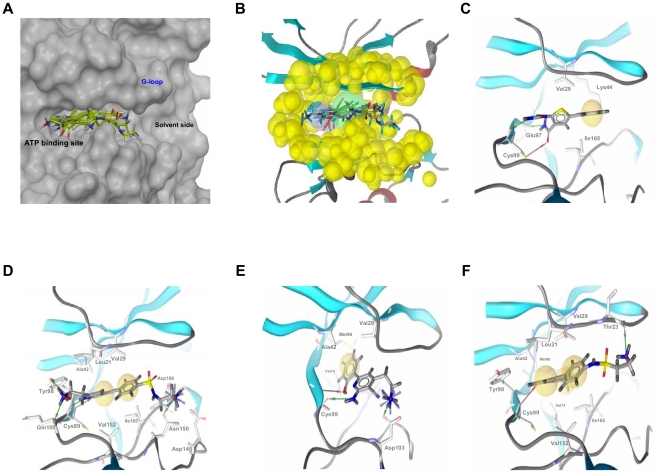
The ATP-binding site, the structure-based common pharmacophore and the best poses for different hIKK-2 inhibitors. The ATP-binding site (Figure 2A), the structure-based common pharmacophore (Figure 2B) and the best poses of the various chemical hIKK-2 inhibitors that were used during the homology-model validation and the structure-based common pharmacophore construction (Figures 2C, 2D, 2E and 2F). In Figures 2C, 2D, 2E and 2F, hydrogen-bond donors, hydrogen-bond acceptors and hydrophobic interactions are shown as red arrows, green arrows and yellow spheres, respectively. Figure 2A shows the ATP-binding pocket of our hIKK-2 homology model and the best poses obtained when docking it with the hIKK-2 inhibitors **13** [27]; **12** [18]; **4a** [23] and **14** [18]. Figure 2B shows the locations on the ATP-binding site of the sites that form the structure-based common pharmacophore developed in the present work and the shell of excluded volumes (in yellow) that schematically represents the locations of the surrounding residues. This pharmacophore is formed by two hydrogen-bond donors (in blue), one hydrogen-bond acceptor (in red) and one hydrophobic region (in green), with tolerances (*i.e.*, radii) of 1.5, 1.5 and 3.0 Å, respectively. Figures 2C, 2D, 2E and 2F show the intermolecular interactions between hIKK-2 and the best poses from inhibitors **13**, **12**, **4a** and **14**, respectively. Figures 2A and 2B were drawn with Maestro v9.0.211 (Schrödinger LLC., Portland, USA; http://www.schrodinger.com), whereas Figures 2C, 2D, 2E and 2F were drawn with LigandScout v2.03.

Finally, the quality of this homology model was tested by using it as a rigid receptor in protein-ligand docking experiments with known hIKK-2 inhibitors and evaluating whether the docking poses reproduced the interactions described in the literature as important for kinase inhibitors, specifically, those with key residues in the hIKK-2 ATP binding pocket [13], [14], [44], [45], [53], [58], [59]. Thus, 36 known inhibitors of hIKK-2 (listed in Table S1) were docked with our hIKK-2 homology model without imposing constrains that forced poses to make specific intermolecular interactions with the target. Next, the resultant hIKK-2 complexes were analyzed with the help of LigandScout to determine which complexes exhibit target-inhibitor intermolecular interactions equivalent to those described in prior studies [14], [15], [31], [44]. This knowledge-based analysis enabled us to find at least one knowledge-based coherent pose for 21 out of the 36 hIKK-2 inhibitors assayed (regardless of their eHiTS scores). Therefore, we concluded that **(a)** our best homology model correctly describes the ATP binding pocket in hIKK-2 (see Figures 2A and 2B) and **(b)** our protein-ligand docking strategy (using the hIKK-2 homology model as a rigid body and only allowing rotation around ligand single bonds of the ligand) is valid for describing the interactions between a large number of known selective ATP-competitive hIKK-2 inhibitors from different chemical families and this target. Notably, this fast protein-ligand docking strategy is appropriate for use in a VS workflow, where other more sophisticated and accurate protein-ligand docking strategies, such as induced-fit docking, are inapplicable [44].

Figures 2C, 2D, 2E and 2F display the knowledge-based coherent docking poses for four selected hIKK-2 inhibitors [*i.e.*, **13** [27] (Figure 2C), **12** [18] (Figure 2D), **4a** [23] (Figure 2E) and **14** [18] (Figure 2F)] that **(a)** belong to three different chemical families [inhibitors **14** [18] (Figure 2F) and **12** [18] (Figure 2D) share the same structural scaffold], **(b)** have different types of intermolecular interactions with the target and **(c)** share some of the hIKK-2 residues with which they interact (for instance, all of them have a hydrogen-bond interaction with the Cys99 backbone and a hydrophobic interaction with Val29). Thus, the analysis of this figure revealed the following:

- Inhibitor **13** [27] (see Figure 2C) is a thiophene carboxamide with substituted ureas that exhibits the most fundamental intermolecular interactions with the target: **(a)** hydrogen bonding between the urea groups and the main-chain oxygens from Glu97 and Cys99 [27], [28], [31] in the hinge region (and also between the carbonyl oxygen atom from **13** and the thiol group from Cys99) and **(b)** hydrophobic interaction with residues Val29, Lys44 and Ile165.
- Inhibitor **12** [18] (see Figure 2D) is an azaindole sulfonamide that exhibits more hydrogen bonds with hIKK-2 than the other inhibitors. Specifically, it has **(a)** two hydrogen bonds between the two nitrogen atoms from the azaindole ring and the main-chain N and O from Cys99 (in agreement with the results of *Liddle et al.* [18]), **(b)** one hydrogen bond between its amide group and the side-chain hydroxyl group of Tyr98, **(c)** one hydrogen bond between the nitrogen from the amide group and the backbone oxygen atom of Gln100, and **(d)** one hydrogen bond on the other side of the binding pocket with the Asn150 and Asp166 side chains. Here, we note that the relevance of Cys99 and Gln100 in this intermolecular interaction has been reported [18]. Furthermore, inhibitor **12** has hydrophobic interactions with the Leu21, Val29, Ala42, Asp145, Val152 and Ile165 side chains.
- Inhibitor **4a** [23] (Figure 2E) is a pyridine derivate that has a very different chemical scaffold than the other inhibitors studied and, moreover, it belongs to a family of very active hIKK-2 inhibitors. The Cys99 backbone atoms are involved in two hydrogen-bonding interactions, one of which is between the nitrogen and the hydroxyl group in the 2′ position of the benzyl moiety of **4a** (which is an important group for this family of hIKK-2 inhibitors [22]–[24], [37]) and the other is between the carbonyl oxygen and one of two amine group of **4a**. Asp103 also contributes to stabilizing inhibitor **4a** on the other side of the ATP pocket (close to the solvent-accessibility region of the binding site) by forming another hydrogen bond between its side-chain oxygen, which accepts the hydrogen from the second **4a** amine group. The theoretical complex also shows that Val29, Ala42, Val74 and Met96 have hydrophobic interactions with the **4a** benzyl ring.
- Inhibitor **14** [18] (Figure 2F) and inhibitor **12** (Figure 2D) share the same structural scaffold but have different residue interactions with the hIKK-2 homology model. Thus, only one residue in the hinge region (Cys99) forms a hydrogen bond with **14** (instead of the three hydrogen bonds made by **12**; see above). Here, the hydrogen bond is between the Cys99 carbonyl oxygen and the nitrogen from the azaindole six-membered ring of **14**. Additionally, the amine group of **14** forms a hydrogen bond with the Thr23 hydroxyl group (located in the G-loop). The hydrophobic interactions of **14** are with Leu21, Val29, Ala42, Val74, Met96, Tyr98, Val152 and Ile165.

### Structure-based pharmacophore description

**Figure 3:**
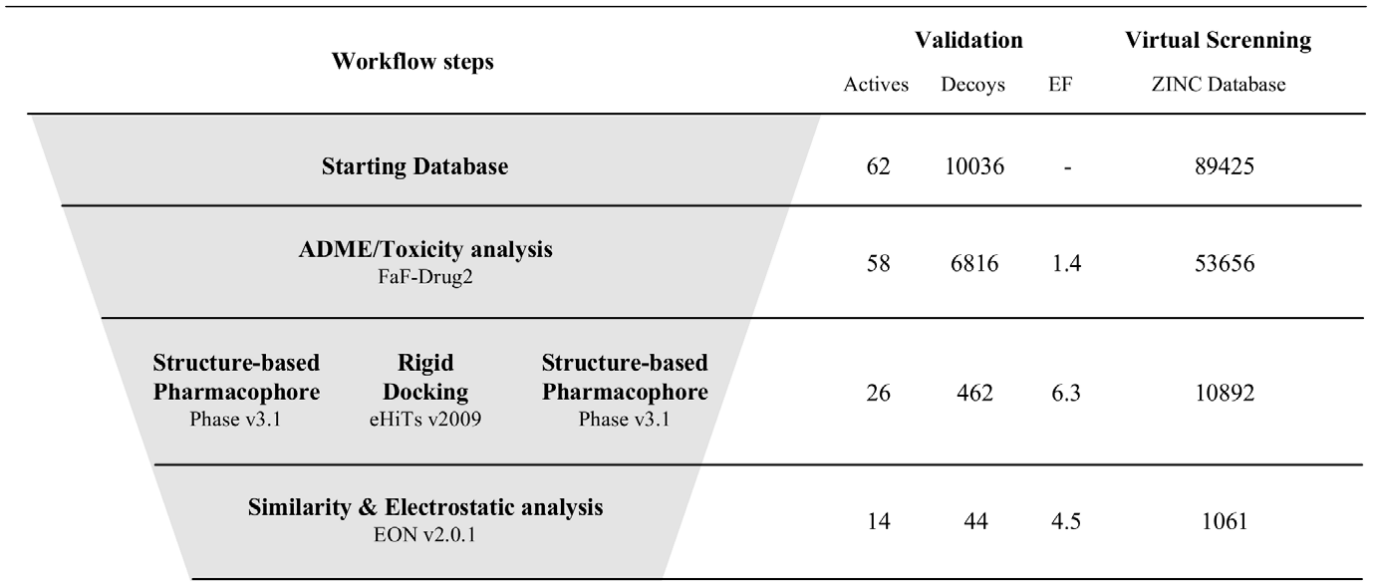
The VS workflow used in the present work. The data beside each VS step show the number of molecules that *survived* it. The *ACTIVES* and *DECOYS* columns refer to known hIKK-2 and kinase decoys used during VS validation, respectively. The *ZINC NPs* column refers to data obtained with the ZINC NP subset (http://wiki.compbio.ucsf.edu/wiki/index.php/Natural_products_database). Enrichment factors were calculated during the validation of each step of the VS protocol as the quotient between the fraction of actives in the sample that survived the VS step and the fraction of actives in the sample before the VS step.

The validation of the homology model for hIKK-2 showed that our protein-ligand docking strategy found 43 knowledge-based coherent poses for 21 out of the 36 hIKK-2 inhibitors assayed. Then, a visual inspection of the chemical features involved in the intermolecular interactions between hIKK-2 and these 43 poses was used to identify those common to most of them and which, therefore, could be used to build a common pharmacophore describing the mechanism of the ligand-target interaction. The resulting common structure-based pharmacophore, shown in Figure 2B, contains one hydrogen-bond acceptor, two hydrogen-bond donors and one hydrophobic site that were common to most of the 43 poses. The spatial location of the hydrogen-bond donors and acceptor sites was nearly coincident for all poses, but this was not the case for the hydrophobic site that occupies a large volume in the ATP-binding site. Thus, in the context of the common structure-based pharmacophore, this was translated into a larger radius for the hydrophobic feature (*i.e.*, 3.0 Å) than for the acceptor and donor radii (*i.e.*, 1.5 Å), as a consequence of the huge structural variability found for the inhibitor moieties that bound to the narrow hydrophobic pocket located between the two hIKK-2 lobes (see Figure 2A). Moreover, to increase the power of the pharmacophore to discriminate between hIKK-2 inhibitors and inactive ligands that could adopt poses that accomplish the pharmacophore features, a shell of excluded volumes was added that schematically represents the locations of the residues forming the ATP-binding site in our hIKK-2 homology model (see Figure 2B). These excluded volumes were especially useful during the second step of the VS workflow (see Figure 3), i.e., the identification of molecules having at least one conformer that, irrespective of its initial spatial orientation, can fit into the pharmacophore (because this shell can be used to exclude from the VS those inactive molecules that have at least one conformer that matches the sites in the pharmacophore but is sterically hindered by other residues in the binding site).

### Virtual-screening workflow: validation and application to the NP subset of the ZINC database

The capacity of our VS workflow to distinguish between hIKK-2 inhibitors and molecules that do no inhibit hIKK-2 was evaluated by applying it to a set consisting of 62 known hIKK-2 inhibitors (different from the 36 used during the *structure-based* pharmacophore generation; see Table S2) and 10,036 kinase decoys obtained from the Directory of Useful Decoys (DUD; http://dud.docking.org) [46]. Figure 3 shows how many actives and decoys *survived* each VS step. Thus, the ADME/Tox, the pharmacophore-docking-pharmacophore and the shape and electrostatic-potential comparison filters produced enrichment factors of 1.4, 6.3 and 4.5, respectively, with a global enrichment factor of 39.3 for the full VS process. Therefore, these results show that our VS protocol is sufficiently selective to discern between those molecules that can inhibit hIKK-2 from those that do not affect its activity. Consequently, it was used to predict that 1,061 out of the 89,425 molecules in the ZINC Natural Products Database were potential hIKK-2 inhibitors (see Figure 3).

### Finding new scaffolds for hIKK-2 inhibitors

**Figure 4:**
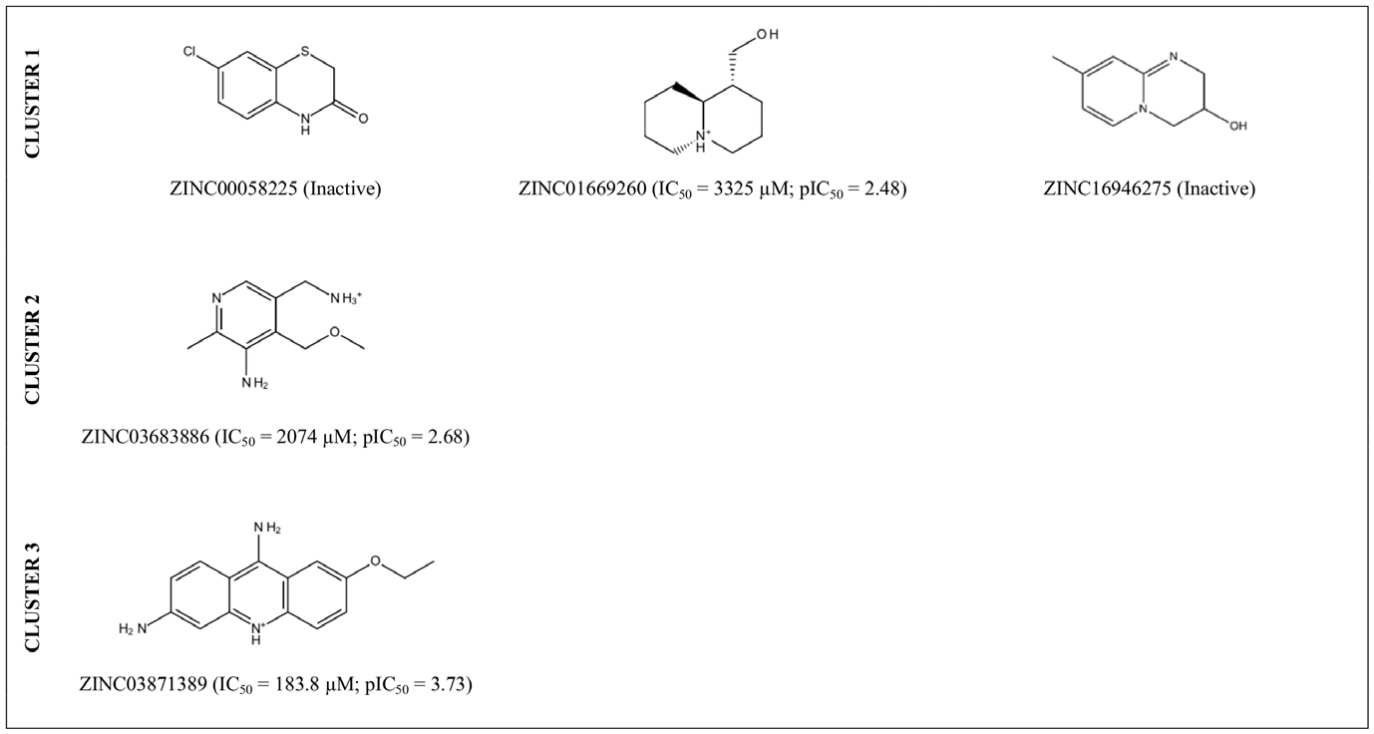
2D structures of the VS hits for which hIKK-2-inhibition activity was tested in an *in vitro* assay. The IC_50_ and pIC_50_ values are also shown for those molecules that inhibit hIKK-2 and were calculated with GraphPad Prism v4.0.

**Figure 5:**
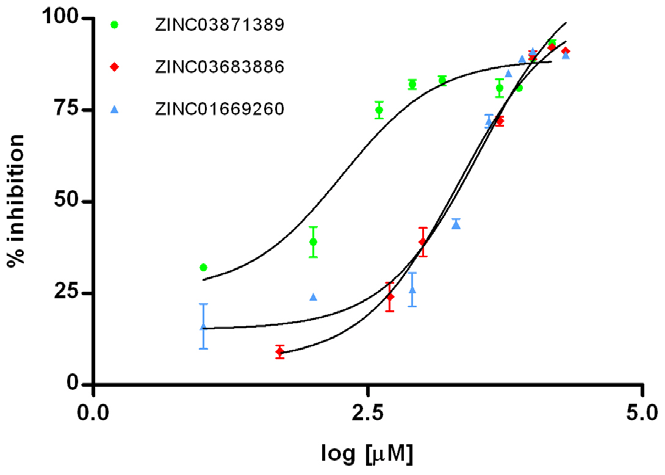
Dose-response results for the *in vitro* inhibition of hIKK-2 by ZINC01669260, ZINC03683886 and ZINC03871389 (with n = 3). The figure was drawn with GraphPad Prism v4.0.

**Figure 6:**
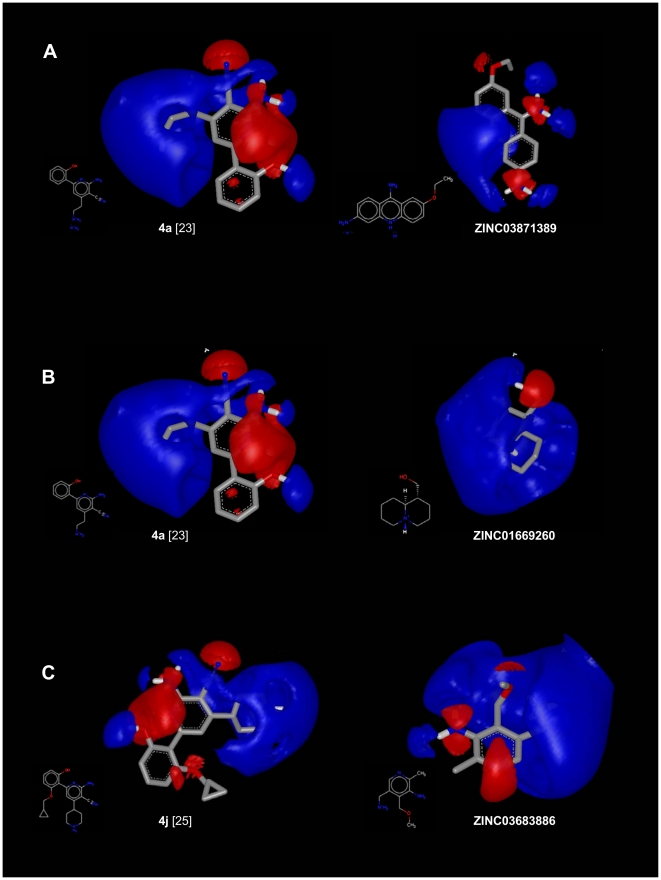
EON results for the best comparison between known hIKK-2 inhibitors and ZINC01669260, ZINC03683886 and ZINC03871389. The best electrostatic-potential and shape comparisons between the docked poses from ZINC01669260, ZINC03683886 and ZINC03871389 and those from the known hIKK-2 inhibitors used by EON as the queries of the last VS filter. For clarity, only the electrostatic potential fields are shown. This Figure was drawn with VIDA v4.03 (OpenEye Scientific Software, Inc., Santa Fe, New Mexico, USA; http://www.eyesopen.com).

One of the most important challenges of any VS workflow is its ability to find molecules with the required activity but without trivial similarity (in terms of chemical structure) to known active compounds. Thus, to determine which of the 1,061 potential hIKK-2 inhibitors predicted by our VS workflow could be considered as new lead molecules that may be of use in drug design and development, we merged these 1,061 NPs with the 98 known hIKK-2 inhibitors used either for validation or for pharmacophore-generation purposes, and the resulting set was classified into clusters (data not shown). Notably, three out of the ten clusters obtained consisted exclusively of NPs (*i.e.*, Clusters 1, 2 and 3 contain 120, 88 and 38 NPs, respectively) and, therefore, these 246 molecules are scaffold-hopping candidates for hIKK-2 inhibition (see Table S3). To prove the reliability of our predictions, we selected 5 of these 246 molecules (ZINC00058225, ZINC01669260 and ZINC16946275 from Cluster 1; ZINC03683886 from Cluster 2; and ZINC03871389 from Cluster 3; see Figure 4) and tested their effects on the hIKK-2 activity using an *in vitro* assay (see Figure 5). The results of this experiment showed that three out of the five molecules (*i.e.*, ZINC01669260 from Cluster 1, ZINC03683886 from Cluster 2 and ZINC03871389 from Cluster 3) inhibited hIKK-2, with IC_50_ values ranging from 183.8 to 3,325 µM. Thus, the highest inhibition rate achieved for ZINC03871389 (ethacridine, ethodin or rivanol) was 93% at a 15-mM inhibitor concentration. Similarly, the highest inhibition rates achieved for ZINC01669260 (lupinine) and ZINC03683886 were 91 and 92% at 10- and 15-mM inhibitor concentrations, respectively. Remarkably, a SciFinder search (Chemical Abstracts Service, Columbus, Ohio, USA; http://www.cas.org/products/sfacad) of the literature in which the three active molecules are cited revealed that none of them have been described as anti-inflammatory drugs. Therefore, this result supports the ability of the electrostatic-potential and shape comparison performed by EON to smooth differences in chemical structures and translate them into criteria important for their intermolecular interactions with the ligand-binding site (see Figure 6).

### Structural analysis of the inhibition of hIKK-2 by ZINC01669260, ZINC03683886 and ZINC03871389

**Figure 7:**
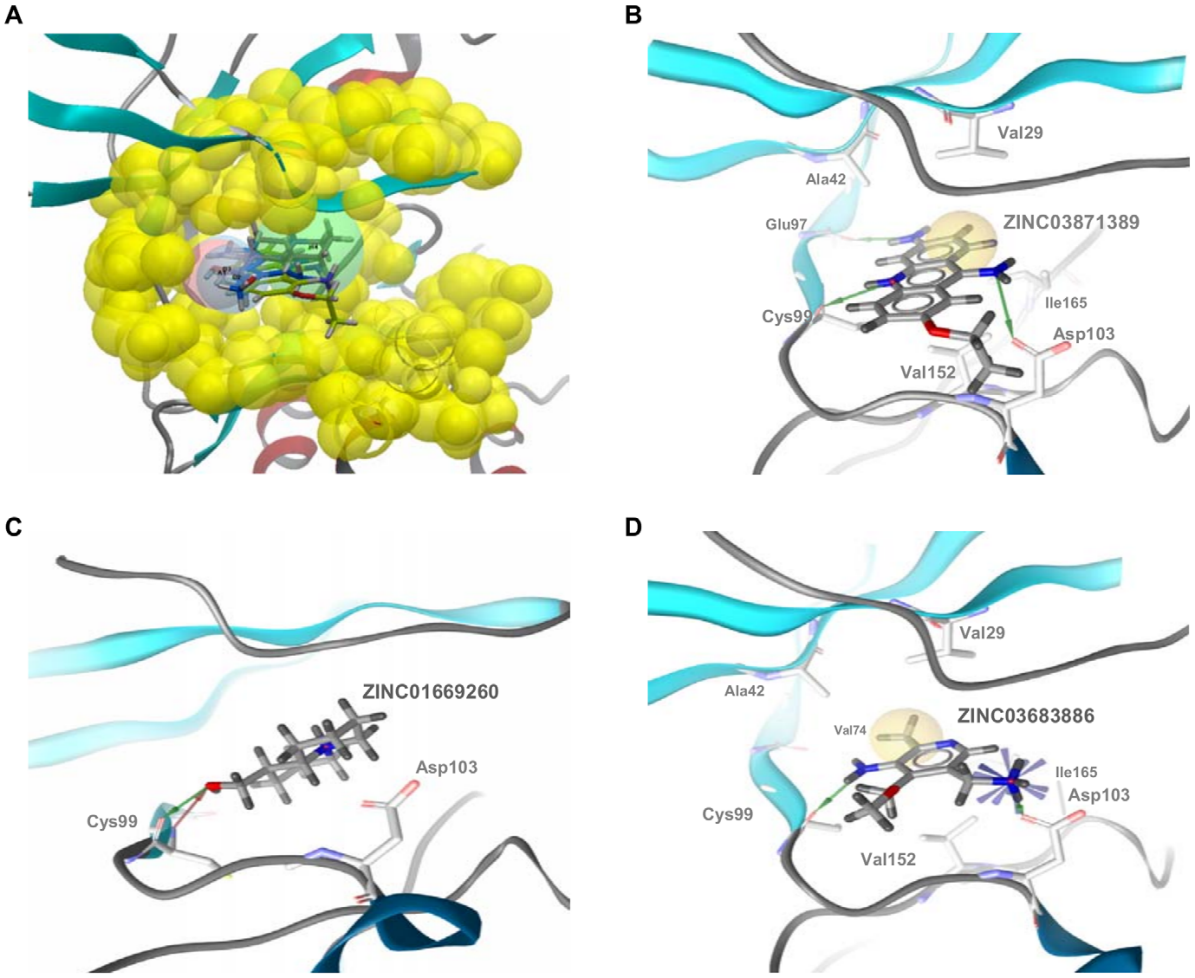
Glide XP results for ZINC01669260, ZINC03683886 and ZINC03871389. Glide XP results for ZINC01669260, ZINC03683886 and ZINC03871389 in the context of the structure-based common pharmacophore (panel A) and the hIKK-2 ATP-binding site (panels B, C and D). Thus, Figure 7A shows that Glide XP was able to find at least one pose for ZINC01669260, ZINC03683886 and ZINC03871389 that matched the pharmacophore. The intermolecular interactions of these poses with the hIKK-2 homology model are shown in Figure 7B (ZINC03871389), 7C (ZINC01669260) and 7D (ZINC03683886). Hydrophobic contacts and hydrogen-bond donors/acceptors are shown as yellow spheres and red/green arrows, respectively.

The docking of ZINC01669260, ZINC03683886 and ZINC03871389 in the ATP-binding site of our hIKK-2 model showed that the three molecules share the same form of intermolecular interaction with the enzyme because they match the structure-based common pharmacophore in the same orientation (see Figure 7A) and because they form a hydrogen bond with the carbonyl oxygen of the gk+3 residue (*i.e.*, Cys99; see Figure 7B, 7C and 7D; Cys99 also uses its backbone nitrogen to make a second hydrogen bond with ZINC01669260; Figure 7C). Moreover, ZINC03871389 and ZINC03683886 also interact with the side-chain oxygen from Asp103 (see Figure 7B and 7D), and only ZINC03871389 is able to form a third hydrogen bond with the main-chain oxygen from the gk+1 residue (*i.e.*, Glu97; see Figure 7B). With respect to the hydrophobic interactions, they were only detected for ZINC03871389 and ZINC03683886 (see Figure 7B and 7D) and involve the acridine moiety of ZINC03871389 (which interacts with Val29, Ala42, Val152 and Ile165) and the methyl group from ZINC03683886 (which interacts with Val29, Ala42, Val74, Val152 and Ile165). Notably, our results show that there is a direct correlation between the number and strength of intermolecular interactions and the inhibitory activity of the three molecules. Thus, ZINC03871389, ZINC03683886 and ZINC01669260 make three/four, two/five and one/zero hydrogen bonds/hydrophobic interactions with key residues on the hIKK-2 ATP binding pocket, and their corresponding IC_50_ values are 183.8, 2,074 and 3,325 µM, respectively.

### Conclusions

The challenge of any VS protocol consists of using *in silico* tools to predict which of the molecules in a database have the required activity against a specific target. Thus, the results of the present study demonstrate that our VS protocol successfully identified hIKK-2 inhibitors with no chemical-structure similarities to known actives and, therefore, it is suitable for scaffold hopping on this target. Moreover, this is the first time that anti-inflammatory activity has been described for ZINC03683886, lupinine (*i.e.*, ZINC01669260) and ethodin (*i.e.*, ZINC03871389; a molecule that is known to cause uterine contractions [59], [60] and that has been also described as inhibitor of the human carboxylesterase 1 [61]). In this sense, the results of the present study outperform those of a recent *in silico* survey on the ChemDiv database (http://us.chemdiv.com) that found hIKK-2 inhibitory activity for only two out of 29 hits selected for *in vitro* testing the VS workflow success rate [42]. Moreover, one of these two molecules shares the same core scaffold with BMS-345541 (a well-known hIKK-2 inhibitor) [62] and, therefore, only the other molecule can be considered a lead compound for the development of new hIKK-2 inhibitors.

Furthermore, although the pIC_50_ of the three hit molecules that showed *in vitro* activity is significantly lower than that for most known hIKK-2 inhibitors used in the present study (see Tables S1 and S2), it is important that these three molecules **(a)** can be used as lead compounds for developing more potent inhibitors using structural-activity relationship studies and **(b)** were selected based on their commercial availability, cost and purity and with the primary goal of testing the performance of our VS protocol. Therefore, it is possible that there are other molecules in the remaining 241 included in Clusters 1, 2 and 3 (see Table S3) that show higher activity towards hIKK-2 than ZINC03871389, ZINC03683886 and ZINC01669260 and could be better lead compounds for drug development than these three molecules. In this sense, our results indicate that the activity correlates well with the *ET_combo* score provided by EON (see Figure 4 and Table S3) and, therefore, this finding suggests that molecules with *ET_combo* values higher than 1.2 (*e.g.*, ZINC12410246, ZINC00485744 and ZINC00071662 from Clusters 1, 2 and 3, respectively) are optimal starting points for the rational drug design of potent and selective hIKK-2 inhibitors with new chemical scaffolds.

### Supporting information

Details on hIKK-2 synthetic inhibitors used in the present work are described in Table S1 (for pharmacophore-generation purposes) and S2 (for validation purposes). Table S3 reports the ZINC codes for the 246 hit molecules that are predicted to inhibit hIKK-2 and that are scaffold-hopping candidates for hIKK-2 inhibition, because the Tanimoto similarities between their MOLPRINT 2D fingerprints and those from the hIKK-2 inhibitors in Table S1 and S2 are significantly low.

## Methods

### Homology-model building of the 3D-structure for the kinase domain of hIKK-2

The sequence of hIKK-2 was obtained from the UniProt database (http://www.uniprot.org, accession number O14920). The protein templates used to build its homology model were PDB structures that simultaneously satisfied the following criteria: **(a)** highest sequence similarity with hIKK-2, **(b)** X-ray-derived structures with acceptable quality (*i.e.*, resolution <2.50 Å and R-value<0.210 Å), **(c)** complexation with inhibitors that bind to the ATP-binding site (because we were interested in the activated form of our templates), **(d)** closest proximity to hIKK-2 [63] in the human kinome tree (although IKK-2 is a serine/threonine kinase without a defined family in this tree), and **(e)** structures with no mutations in the active site of the kinase domain. The two kinases that fulfilled these criteria and were thus chosen as templates belong to the CAMK kinase family [63], have a 32% sequence identity with the kinase domain of hIKK-2, have a 50% identity with the residues of the ATP-binding site of hIKK-2 (where these residues correspond to those in the G-loop, hinge region, HRD motif and catalytic loop); they are the serine/threonine-protein kinase CHK2 (PDB code 2CN5 [56]) and the death-associated protein kinase 3 or ZIP kinase (PDB code 2J90 [57]). The superposition of both template structures revealed some differences in their loop structures: **(a)** the G-loop is more open in 2J90 than in 2CN5 and **(b)** the A-loop coordinates were not present for 2J90. Armed with this information and following a knowledge-based approach, we used MODELLER v9.5 [64] to align the sequences and construct several models by changing their alignments with the kinase domain of hIKK-2. The resulting models were subsequently refined with MODELLER in a two-step process (*i.e.*, loops were refined first without the use of the templates following an *ab initio* methodology [65], and α-helices were the refined when loop processing was finished using the templates) and using default running conditions. Next, the quality of the resulting models was evaluated by **(a)** assessing the packing quality with the **A**tomic **No**n-**L**ocal **E**nvironment **A**ssessment (ANOLEA) webserver (http://protein.bio.puc.cl/cardex/servers/anolea) [66], **(b)** analyzing the outliers in the Ramachandran plot; **(c)** examining the ATP-binding site structure (because it must be optimized to built a receptor-based pharmacophore), and **(d)** performing a protein-ligand rigid-docking study with the eHiTS v2009 [67] software (SimBioSys Inc., Toronto, Canada; http://www.simbiosys.ca/ehits) using various known hIKK-2 inhibitors[47], [48] and visually analyzing the knowledge-based coherence of the results. In this sense, the capability of eHiTS to predict a knowledge-based coherent pose was assessed by redocking the two complexes used as templates to obtain the homology models. Thus, eHiTS predicted poses for the ADP and pyridone 6 with root-mean-square deviations relative to their experimental poses in 2CN5 and 2J90 of 1.23 and 0.38 Å, respectively.

### Structure-based pharmacophore generation

The best homology model obtained for the hIKK-2 kinase domain was subsequently used as the receptor in a protein-ligand docking with 36 known selective ATP-competitive inhibitors for hIKK-2 from different chemical families (see Table S1) [10], [11], [13], [15], [16], [18], [20]–[31]. The docking was done with eHiTS v2009 [67] by considering the receptor to be a rigid body and the ligands as flexible (*i.e.*, free rotation was allowed around the single bonds of the ligand). Default docking conditions were selected except for the size of the sides of the cubic box encompassing the ATP-binding pocket, which was increased from 10 Å to 15 Å. Thereafter, all subsequent protein-ligand dockings in this work, either for validation or VS purposes, were performed with eHiTS v2009 using the same running conditions, hIKK-2 area and homology-model orientation. Glide XP v5.6 (Schrödinger LLC., Portland, USA; http://www.schrodinger.com), running with default conditions, was used to predict the poses of three NPs that our VS workflow predicted as hIKK-2 inhibitors and for which activity was confirmed by the *in vitro* assay.

The protein-ligand interactions of the poses obtained by the docking process (a maximum of 32 poses per ligand) were analyzed with LigandScout v2.03 (Inte:ligand GmbH, Vienna, Austria, http://www.inteligand.com/ligandscout) [68]. Next, the poses that satisfied the generic binding features of ATP-competitive kinase inhibitors [45] [*i.e.*, the formation of hydrogen bonds with the main chain of residues in the hinge region (segment 96–99 in hIKK-2 sequence) and hydrophobic interactions with the hydrophobic pocket of hIKK-2 (*e.g.* Val29, Lys44, Val152, and Ile65)] were selected as knowledge-based coherent (regardless of their eHiTS scores). As a result of this analysis, 43 poses of the 21 hIKK-2 inhibitors were identified as knowledge-based coherent, and their corresponding sites (*i.e.*, functional groups used by the poses in their intermolecular interactions with the hIKK-2 kinase domain) were used to derive a common structure-based pharmacophore. This pharmacophore contains one hydrogen-bond acceptor, two hydrogen-bond donors and one hydrophobic site common to most of the 43 poses. Moreover, the pharmacophore was completed with a shell of excluded volumes that schematically represents the location of the residues forming the ATP-binding site in our hIKK-2 homology model. This shell was built by applying the *Receptor-Based Excluded Volumes* graphic front-end in Phase v3.1 (Schrödinger LLC., Portland, USA; http://www.schrodinger.com) [69] to our hIKK-2 kinase domain model and by setting the *Sphere filters* parameter values to **(a)** *ignore receptor atoms with surfaces within* ***0.25*** *Å of the ligand surface* and **(b)** *limit excluded-volume shell thickness to* ***10*** *Å*. The remaining parameters used in the front-end were the defaults.

### Ligand setup

All the ligands used in this work (either for validation methodology or for VS purposes) were processed in the same way. The 3D ligand structures were either downloaded from public databases [*i.e.*, DUD [46], the BindingDB (http://www.bindingdb.org) [47] and the ZINC Natural Products Database (http://wiki.compbio.ucsf.edu/wiki/index.php/Natural_products_database) [48]] or constructed with ChemDraw Ultra v11.0 (CambridgeSoft Corporation, Cambridge, MA, USA; http://www.cambridgesoft.com) [70]. These 3D structures were then incorporated into LigPrep v2.3 (Schrödinger LLC., Portland, USA; http://www.schrodinger.com) and improved by cleaning. The cleaning process was carried out with the following parameters: **(a)** the force field used was OPLS 2005; **(b)** all possible ionization states at pH 7.0±2.0 were generated with Ionizer; **(c)** the *desalt* option was activated; **(d)** tautomers were generated for all ionization states at pH 7.0±2.0; **(e)** chiralities, when present, were determined from the 3D structure; and **(f)** one low-energy ring conformation per ligand was generated. Conformations and sites for the resulting ligand structures were determined during the generation of the corresponding Phase [69] databases with the *Generate Phase Database* graphic front-end. The parameter values used during this conformer generation were by default with the exception of the maximum number of conformers per structure, which was increased from 100 (the default value) to 200. The conformer sites were generated with definitions made by adding the ability to consider aromatic rings as hydrophobic groups to the default built-in Phase definitions.

### Virtual-screening workflow

The NP subset of the ZINC database (http://zinc.docking.org/vendor0/npd) [48] contains the 3D structure of 89,425 commercially available NPs and NP derivatives and was used as the source of molecules to which our VS schema was applied in the search for new hIKK-2 inhibitors. Initially, these 89,425 molecules were submitted to an ADME/Tox filter with the FAF-Drugs2 tool [71], which aimed to discard molecules that either had poor ADME properties or were potentially toxic. Thus, the drug-like properties of a compound were evaluated using the Lipinski rule [72], and only one violation of this rule was allowed. This rule is based on a set of property values (*i.e.*, the number of hydrogen-bond donors and acceptors, the molecular weight and the logP) that were derived from drugs with good ADME characteristics [72]. Therefore, molecules that pass the Lipinski rule are expected to be active in humans after oral admission. Moreover, molecules containing toxic groups were filtered using the 204 substructures for “warhead” chelators, frequent hitters, promiscuous inhibitors and other undesirable functional groups available in FAF-Drugs2 tool [71].

Molecules with appropriate ADME/Tox properties were then set up with LigPrep and used to generate a Phase database. Next, this database was filtered with Phase through the structure-based common pharmacophore using the following running conditions: **(a)** search in the conformers database, **(b)** do not *score in place* the conformers into the structure-based common pharmacophore (*i.e.*, allow reorientation of the conformers to determine if they match the pharmacophore or not), **(c)** match at least three out of the four sites of the structure-based common pharmacophore, **(d)** do not consider any site as mandatory, **(e)** do not prefer partial matches involving more sites and **(f)** use the excluded volumes from the structure-based common pharmacophore. The rest of options and parameter values used during that search were the default values.

Those ligands with at least one hit in this Phase search were then docked to our hIKK-2 homology model. Then, the resulting ligand poses were again filtered with Phase through the structure-based common pharmacophore using the same filtering conditions as in the first Phase run, with the exception that no reorientation of the poses was allowed during the search (*i.e.*, the *score in place* option was used) to find docking poses that were compatible with the pharmacophore.

Finally, the poses that were hits in this second pharmacophore screening were submitted to a shape and electrostatic-potential comparison with 43 poses from 21 known hIKK-2 inhibitors (see Table S1) that **(a)** were also obtained by docking with our hIKK-2 homology model and **(b)** also matched the structure-based common pharmacophore without reorientation. This comparison was done with EON v2.0.1 (OpenEye Scientific Software, Inc., Santa Fe, New Mexico, USA; http://www.eyesopen.com) using the *ET_combo* score as the similarity criterion. The *ET_combo* is the sum of two calculations: **(a)** the *Shape Tanimoto*, which is a quantitative measure of three-dimensional overlap [where 1 corresponds to a perfect overlap (*i.e., the* same shape)] [73] and **(b)** the *Poisson-Boltzman Electrostatic Tanimoto*, which compares the electrostatic potential of two small molecules (where 1 corresponds to identical potentials and negative values correspond to the overlap of positive and negative charges) [74]. Only those NPs with *ET_combo* values equal to or greater than 0.850 were considered as candidates for experimental tests of their activity on hIKK-2.

### Virtual-screening workflow validation

The ability of the VS workflow to identify hIKK-2 inhibitors in a database of molecules was tested by applying the same filtering method described for the NPs (*i.e., the* same methodology and parameter values) to: **(a)** a group of 62 known inhibitors different from the 36 used for the *structure-based* pharmacophore generation (see Table S2) and **(b)** an external group of 10,139 kinase decoys for Cdk2 and p38 (CMGC family at the human kinome tree [63]) that were obtained from DUD [46].

### Hit selection for further experimental assays on hIKK-2 activity

A structural-similarity analysis of the NPs with *ET_combo* values equal to or greater than 0.850 and all known hIKK-2 inhibitors used in this work (either for validation or for pharmacophore-generation purposes) was performed by executing a Schrödinger script that clusters molecules based on Tanimoto similarities between MOLPRINT 2D fingerprints. We obtained ten clusters and, notably, three of them consisted only of NPs. Thus, five compounds from these three clusters were selected [based on their commercial availability, cost and purity (≥95%)] for experimental assay of their effects on hIKK-2 activity. These compounds were ZINC03683886, ZINC00242004 and ZINC03871389, bought from InterBioScreen, Ltd (http://www.ibscreen.com), and ZINC00058225 and ZINC01669260, bought from TimTec, Inc. (http://www.timtec.net).

### *In vitro* assay of the effect of selected compounds on the hIKK-2 activity

The effect of the selected compounds on hIKK-2 kinase activity was determined using the Calbiochem® K-LISA™ IKKβ Inhibitor Screening Kit, designed for rapid *in vitro* screening of IKK-2 inhibitors. Briefly, the kit consists of an ELISA-based activity assay that uses a 50-amino-acid GST-IκBα fusion-polypeptide substrate that includes the Ser^32^ and Ser^36^ IKK-2 phosphorylation sites. The GST-IκBα substrate and IKK-2 are incubated in the presence of IKK-2 inhibitors in the wells of a glutathione-coated 96-well plate, which allows for substrate phosphorylation and capture in a single step. The phosphorylated GST-IκBα substrate is detected using an anti-phospho-IκBα (Ser^32^/Ser^36^) antibody followed by an HRP-conjugate and color development with the TMB substrate. The ELISA stop solution is then used to stop the color development, and the absorbance is read at 450 nm (with a reference wavelength of 540–600 nm). The absorbance is directly related to the IKK-2 activity level. Moreover, following the kit instructions, one positive control and three negative controls were included by varying the absence or presence of the commercial hIKK-2 inhibitor IV (Calbiochem catalogue number: 401481), the ATP/MgCl_2_ mix and the hIKK-2 enzyme. The results of these controls confirmed the absence of nonspecific or artificial inhibition. Three repetitions were made for all the activity assays.

### IC_50_ calculation

The potential of the molecules to inhibit hIKK-2 activity was quantified by calculating their IC_50_ values. This parameter was determined using GraphPad Prism v4.0 for Windows (GraphPad Software, San Diego CA, USA; http://www.graphpad.com) by fitting the experimental data from the *in vitro* assay to a nonlinear regression function using a four-parameter logistic equation.

## Supporting Information

Table S1 hIKK-2 inhibitors from different chemical families docked with the hIKK-2 homology model. These 36 hIKK-2 inhibitors were docked with our hIKK-2 homology model without imposing constraints that forced poses to make specific intermolecular interactions with the target. Next, the resulting hIKK-2 complexes were analyzed with the help of LigandScout to determine which complexes exhibited target-inhibitor intermolecular interactions equivalent to those described in prior studies. This knowledge-based analysis enabled us to identify at least one knowledge-based coherent pose (43 in total) for 21 out of the 36 hIKK-2 inhibitors assayed (regardless of their scoring by eHiTS; see their values in the *Knowledge-based coherent docking poses* column). By analyzing the chemical features used by each pose in its intermolecular interaction with hIKK-2, a common pharmacophore was derived that describes the mechanism of the ligand-target interaction. The *Cluster* column shows the cluster in which each molecule was classified after running a Schrödinger script that clusters molecules based on Tanimoto similarities between MOLPRINT 2D fingerprints (using the Knime v.2.0.3 module in the Schrödinger software package). The molecules distributed in these clusters are the natural products obtained as hits in our virtual-screening protocol and all known hIKK-2 inhibitors used in the present work [either for validation (see Table S2) or for pharmacophore-generation purposes]. The pIC_50_ values were obtained from the literature.
(PDF)

Table S2 hIKK-2 inhibitors used during the validation of the virtual-screening workflow. These 62 hIKK-2 inhibitors (different from the 36 used during the structure-based pharmacophore generation; see Table S1) were used to test the ability of the virtual-screening workflow to identify hIKK-2 inhibitors in a database of molecules. The *Cluster* column shows the cluster into which each molecule was classified after running a Schrödinger script that clusters molecules based on Tanimoto similarities between MOLPRINT 2D fingerprints (using the Knime v.2.0.3 module in the Schrödinger software package). The molecules distributed in these clusters are the natural products obtained as hits in our virtual-screening protocol and all known hIKK-2 inhibitors used in the present work (either for validation or for pharmacophore-generation purposes). The pIC_50_ values were obtained from the literature.
(PDF)

Table S3 Scaffold-hopping candidates for hIKK-2 inhibition predicted by our study. The ZINC codes for the 246 hit molecules predicted to inhibit hIKK-2 and belonging to clusters consisting exclusively of natural products. For each hit molecule, the best results of the shape and electrostatic-potential comparisons with 43 poses from 21 known hIKK-2 inhibitors (see Table S1) is shown. Thus, the Tanimoto values for the comparison between the electrostatic potentials of the molecules (using an outer dielectric of 80) are shown in the ***PB*** columns, whereas the values for the comparison between shapes are shown in the ***Shape*** columns. The sum of the PB and Shape values is reported in the ***Combo*** columns. Hits from each cluster are sorted according their decreasing combo value. All of these hit molecules are scaffold-hopping candidates for hIKK-2 inhibition because the Tanimoto similarities between their MOLPRINT 2D fingerprints and those from the hIKK-2 inhibitors in Tables S1 and S2 are quite low. ZINC00058225, ZINC01669260 and ZINC16946275 from Cluster 1, ZINC03683886 from Cluster 2, and ZINC03871389 from Cluster 3 were selected to experimentally test the success rate of our predictions using an *in vitro* assay (in bold in Table S3). The results of this experiment showed that three out of the five molecules (*i.e.*, ZINC01669260 from Cluster 1, ZINC03683886 from Cluster 2 and ZINC03871389 from Cluster 3) inhibited hIKK-2, with IC_50_ values ranging from 183.8 to 3,325 µM.
(PDF)

## References

[1] Rollinger JM, Stuppner H, Langer T, Petersen F, Amstutz R (2008). Virtual screening for the discovery of bioactive natural products.. Natural Compounds as Drugs.

[2] Cragg GM, Newman DJ (2009). Nature: a vital source of leads for anticancer drug development.. Phytochem Rev.

[3] Harvey AL (2008). Natural products in drug discovery.. Drug Discov Today.

[4] Rollinger JM, Langer T, Stuppner H (2006). Strategies for efficient lead structure discovery from natural products.. Curr Med Chem.

[5] Kumar K, Waldmann H (2009). Synthesis of Natural Product Inspired Compound Collections.. Angew Chem Int Edit.

[6] Williams PG (2009). Panning for chemical gold: marine bacteria as a source of new therapeutics.. Trends Biotechnol.

[7] Zhou XW, Gong ZH, Su Y, Lin J, Tang KX (2009). Cordyceps fungi: natural products, pharmacological functions and developmental products.. J Pharm Pharmacol.

[8] Rollinger JM, Langer T, Stuppner H (2006). Integrated *in silico* tools for exploiting the natural products' bioactivity.. Planta Med.

[9] Castro AC, Dang LC, Soucy F, Grenier L, Mazdiyasni H (2003). Novel IKK inhibitors: β-carbolines.. Bioorg Med Chem Lett.

[10] Clare M, Fletcher T, Hamper BC, Hanson G, Heier RF (2005). Substituted pyrazole urea compounds for the treatment of inflammation..

[11] Karin M, Yamamoto Y, Wang QM (2004). The IKK NF-κB system: a treasure trove for drug development.. Nat Rev Drug Discov.

[12] Bhagwat SS (2009). Kinase inhibitors for the treatment of inflammatory and autoimmune disorders.. Purinergic Signal.

[13] Nagarajan S, Doddareddy M, Choo H, Cho YS, Oh KS (2009). IKKβ inhibitors identification part I: homology model assisted structure based virtual screening.. Bioorg Med Chem.

[14] Nagarajan S, Choo H, Cho YS, Oh KS, Lee BH (2010). IKKβ inhibitors identification part II: ligand and structure-based virtual screening.. Bioorg Med Chem.

[15] Christopher JA, Avitabile BG, Bamborough P, Champigny AC, Cutler GJ (2007). The discovery of 2-amino-3,5-diarylbenzamide inhibitors of IKK-α and IKK-β kinases.. Bioorg Med Chem Lett.

[16] Bingham AH, Davenport RJ, Gowers L, Knight RL, Lowe C (2004). A novel series of potent and selective IKK2 inhibitors.. Bioorg Med Chem Lett.

[17] Bingham AH, Davenport RJ, Fosbeary R, Gowers L, Knight RL (2008). Synthesis and structure-activity relationship of aminopyrimidine IKK2 inhibitors.. Bioorg Med Chem Lett.

[18] Liddle J, Bamborough P, Barker MD, Campos S, Cousins RP (2009). 4-Phenyl-7-azaindoles as potent and selective IKK2 inhibitors.. Bioorg Med Chem Lett.

[19] Christopher JA, Bamborough P, Alder C, Campbell A, Cutler GJ (2009). Discovery of 6-aryl-7-alkoxyisoquinoline inhibitors of IκB kinase-β (IKK-β).. J Med Chem.

[20] Beaulieu F, Ouellet C, Ruediger EH, Belema M, Qiu YP (2007). Synthesis and biological evaluation of 4-amino derivatives of benzimidazoquinoxaline, benzimidazoquinoline, and benzopyrazoloquinazoline as potent IKK inhibitors.. Bioorg Med Chem Lett.

[21] Belema M, Bunker A, Nguyen V, Beaulieu F, Ouellet C (2007). Synthesis and structure-activity relationship of imidazo(1,2-a)thieno(3,2-e)pyrazines as IKK-β inhibitors.. Bioorg Med Chem Lett.

[22] Murata T, Shimada M, Sakakibara S, Yoshino T, Kadono H (2003). Discovery of novel and selective IKK-β serine-threonine protein kinase inhibitors. Part 1.. Bioorg Med Chem Lett.

[23] Murata T, Shimada M, Kadono H, Sakakibara S, Yoshino T (2004). Synthesis and structure-activity relationships of novel IKK-β inhibitors. Part 2: Improvement of *in vitro* activity.. Bioorg Med Chem Lett.

[24] Murata T, Shimada M, Sakakibara S, Yoshino T, Masuda T (2004). Synthesis and structure-activity relationships of novel IKK-β inhibitors. Part 3: Orally active anti-inflammatory agents.. Bioorg Med Chem Lett.

[25] Ziegelbauer K, Gantner F, Lukacs N, Berlin A, Fuchikami K (2005). A selective novel low-molecular-weight inhibitor of IκB kinase-β (IKK-β) prevents pulmonary inflammation and shows broad anti-inflammatory activity.. Br J Pharmacol.

[26] Waelchli R, Bollbuck B, Bruns C, Buhl T, Eder J (2006). Design and preparation of 2-benzamido-pyrimidines as inhibitors of IKK.. Bioorg Med Chem Lett.

[27] Baxter A, Brough S, Cooper A, Floettmann E, Foster S (2004). Hit-to-lead studies: the discovery of potent, orally active, thiophenecarboxamide IKK-2 inhibitors.. Bioorg Med Chem Lett.

[28] Morwick T, Berry A, Brickwood J, Cardozo M, Catron K (2006). Evolution of the thienopyridine class of inhibitors of IκB kinase-β. Part I: hit-to-lead strategies.. J Med Chem.

[29] Bonafoux D, Bonar S, Christine L, Clare M, Donnelly A (2005). Inhibition of IKK-2 by 2-[(aminocarbonyl)amino]-5-acetylenyl-3-thiophenecarboxamides.. Bioorg Med Chem Lett.

[30] Sommers CD, Thompson JM, Guzova JA, Bonar SL, Rader RK (2009). Novel tight-binding inhibitory factor-κB kinase (IKK-2) inhibitors demonstrate target-specific anti-inflammatory activities in cellular assays and following oral and local delivery in an *in vivo* model of airway inflammation.. J Pharmacol Exp Ther.

[31] Sugiyama H, Yoshida M, Mori K, Kawamoto T, Sogabe S (2007). Synthesis and structure activity relationship studies of benzothieno[3,2-b]furan derivatives as a novel class of IKKβ inhibitors.. Chem Pharm Bull (Tokyo).

[32] Bhattarai BR, Ko JH, Shrestha S, Kafle B, Cho H (2010). Inhibition of IKK-β: A new development in the mechanism of the anti-obesity effects of PTP1B inhibitors SA18 and SA32.. Bioorg Med Chem Lett.

[33] Crombie AL, Sum FW, Powell DW, Hopper DW, Torres N (2010). Synthesis and biological evaluation of tricyclic anilinopyrimidines as IKK β inhibitors.. Bioorg Med Chem Lett.

[34] De Silva D, Mitchell MD, Keelan JA (2010). Inhibition of choriodecidual cytokine production and inflammatory gene expression by selective IκB kinase (IKK) inhibitors.. Brit J Pharmacol.

[35] Shimizu H, Tanaka S, Toki T, Yasumatsu I, Akimoto T (2010). Discovery of imidazo 1,2-b pyridazine derivatives as IKK β inhibitors. Part 1: Hit-to-lead study and structure-activity relationship.. Bioorg Med Chem Lett.

[36] Mbalaviele G, Sommers CD, Bonar SL, Mathialagan S, Schindler JF (2009). A Novel, Highly Selective, Tight Binding IκB Kinase-2 (IKK-2) Inhibitor: A Tool to Correlate IKK-2 Activity to the Fate and Functions of the Components of the Nuclear Factor-κB Pathway in Arthritis-Relevant Cells and Animal Models.. J Pharmacol Exp Ther.

[37] Sala E, Guasch L, Vaqué M, Mateo-Sanz JM, Blay M (2009). 3D-QSAR Study of Pyridine Derivates as IKK-2 Inhibitors.. QSAR Comb Sci.

[38] Berman HM, Battistuz T, Bhat TN, Bluhm WF, Bourne PE (2002). The Protein Data Bank.. Acta Crystallogr D.

[39] Rushe M, Silvian L, Bixler S, Chen LL, Cheung A (2008). Structure of a NEMO/IKK-associating domain reveals architecture of the interaction site.. Structure.

[40] Nagarajan S, Ahmed A, Choo H, Cho YS, Oh KS (2010). 3D QSAR pharmacophore model based on diverse IKKβ inhibitors.. J Mol Model.

[41] Noha SM, Atanasov AG, Schuster D, Markt P, Fakhrudin N (2010). Discovery of a novel IKK-β inhibitor by ligand-based virtual screening techniques.. Bioorg Med Chem Lett.

[42] Nagarajan S, Choo H, Cho YS, Shin KJ, Oh KS (2010). IKKβ inhibitor identification: a multi-filter driven novel scaffold.. BMC Bioinformatics.

[43] Avila CM, Romeiro NC, Sant'Anna CMR, Barreiro EJ, Fraga CAM (2009). Structural insights into IKK β inhibition by natural products staurosporine and quercetin.. Bioorg Med Chem Lett.

[44] Lauria AIM, Fazzari M, Tutone M, Di Blasi F, Mingoia F (2010). IKK-β inhibitors: an analysis of drug-receptor interaction by using Molecular Docking and Pharmacophore 3D-QSAR approaches.. J Mol Graph Model.

[45] Ghose AK, Herbertz T, Pippin DA, Salvino JM, Mallamo JP (2008). Knowledge based prediction of ligand binding modes and rational inhibitor design for kinase drug discovery.. J Med Chem.

[46] Huang N, Shoichet BK, Irwin JJ (2006). Benchmarking sets for molecular docking.. J Med Chem.

[47] Liu TQ, Lin YM, Wen X, Jorissen RN, Gilson MK (2007). BindingDB: a web-accessible database of experimentally determined protein-ligand binding affinities.. Nucleic Acids Res.

[48] Irwin JJ, Shoichet BK (2005). ZINC - A free database of commercially available compounds for virtual screening.. J Chem Inf Model.

[49] Schmid JA, Birbach A (2008). IκB kinase β (IKKβ/IKK2/IKBKB)–a key molecule in signaling to the transcription factor NF-κB.. Cytokine Growth Factor Rev.

[50] Kornev A, Haste N, Taylor S, Eyck L (2006). Surface comparison of active and inactive protein kinases identifies a conserved activation mechanism.. Proc Natl Acad Sci U S A.

[51] Rabiller M, Getlik M, Klüter S, Richters A, Tückmantel S (2010). Proteus in the world of proteins: conformational changes in protein kinases.. Arch Pharm (Weinheim).

[52] Delhase M, Hayakawa M, Chen Y, Karin M (1999). Positive and negative regulation of IκB kinase activity through IKKβ subunit phosphorylation.. Science.

[53] Sowadski JM, Epstein LF, Lankiewicz L, Karlsson R (1999). Conformational diversity of catalytic cores of protein kinases.. Pharmacol Ther.

[54] Engh RA, Bossemeyer D (2002). Structural aspects of protein kinase control-role of conformational flexibility.. Pharmacol Ther.

[55] Eglen R, Reisine T (2009). The current status of drug discovery against the human kinome.. Assay Drug Dev Technol.

[56] Oliver AW, Paul A, Boxall KJ, Barrie SE, Aherne GW (2006). Trans-activation of the DNA-damage signalling protein kinase Chk2 by T-loop exchange.. EMBO J.

[57] Pike AC, Rellos P, Niesen FH, Turnbull A, Oliver AW (2008). Activation segment dimerization: a mechanism for kinase autophosphorylation of non-consensus sites.. EMBO J.

[58] Noble ME, Endicott JA, Johnson LN (2004). Protein kinase inhibitors: insights into drug design from structure.. Science.

[59] Rudolph MI, Cabanillas A, Gomez P, Garcia MA, Villan L (1997). On the mechanism of action of ethodin in inducing myometrium contractions.. Gen Pharmacol.

[60] Rudolph MI, de los Angeles Garcia M, Sepulveda M, Brandan E, Reinicke K (1997). Ethodin: pharmacological evidence of the interaction between smooth muscle and mast cells in the myometrium.. J Pharmacol Exp Ther.

[61] Bencharit S, Morton CL, Hyatt JL, Kuhn P, Danks MK (2003). Crystal structure of human carboxylesterase 1 complexed with the Alzheimer's drug tacrine: from binding promiscuity to selective inhibition.. Chem Biol.

[62] Burke JR, Pattoli MA, Gregor KR, Brassil PJ, MacMaster JF (2003). BMS-345541 is a highly selective inhibitor of IκB kinase that binds at an allosteric site of the enzyme and blocks NF-κB-dependent transcription in mice.. J Biol Chem.

[63] Manning G, Whyte DB, Martinez R, Hunter T, Sudarsanam S (2002). The protein kinase complement of the human genome.. Science.

[64] Eswar N, Webb B, Marti-Renom MA, Madhusudhan MS, Eramian D (2006). Comparative protein structure modeling using Modeller.. Curr Protoc Bioinformatics.

[65] Bonneau R, Baker D (2001). *Ab initio* protein structure prediction: progress and prospects.. Annu Rev Biophys Biomol Struct.

[66] Melo F, Feytmans E (1998). Assessing protein structures with a non-local atomic interaction energy.. J Mol Biol.

[67] Zsoldos Z, Reid D, Simon A, Sadjad BS, Johnson AP (2006). eHiTS: an innovative approach to the docking and scoring function problems.. Curr Protein Pept Sci.

[68] Wolber G, Langer T (2005). LigandScout: 3-D pharmacophores derived from protein-bound Ligands and their use as virtual screening filters.. J Chem Inf Model.

[69] Dixon SL, Smondyrev AM, Knoll EH, Rao SN, Shaw DE (2006). PHASE: a new engine for pharmacophore perception, 3D QSAR model development, and 3D database screening: 1. Methodology and preliminary results.. J Comput Aided Mol Des.

[70] Mills N (2006). ChemDraw ultra 10.0.. J Am Chem Soc.

[71] Lagorce D, Sperandio O, Galons H, Miteva MA, Villoutreix BO (2008). FAF-Drugs2: free ADME/tox filtering tool to assist drug discovery and chemical biology projects.. BMC Bioinformatics.

[72] Lipinski CA, Lombardo F, Dominy BW, Feeney PJ (2001). Experimental and computational approaches to estimate solubility and permeability in drug discovery and development settings.. Adv Drug Deliv Rev.

[73] Rush TS, Grant JA, Mosyak L, Nicholls A (2005). A shape-based 3-D scaffold hopping method and its application to a bacterial protein-protein interaction.. J Med Chem.

[74] Naylor E, Arredouani A, Vasudevan SR, Lewis AM, Parkesh R (2009). Identification of a chemical probe for NAADP by virtual screening.. Nat Chem Biol.

