# Supplementary material for: Identification of Human IKK-2 Inhibitors of Natural Origin (Part I): Modeling of the IKK-2 Kinase Domain, Virtual Screening and Activity Assays
Source: PLoS One. 2011 Feb 24;6(2):e16903. doi: 10.1371/journal.pone.0016903 (PMC3044726; doi:10.1371/journal.pone.0016903)
Supplement: Table S1 — hIKK-2 inhibitors from different chemical families docked with the hIKK-2 homology model. These 36 hIKK-2 inhibitors were docked with our hIKK-2 homology model without imposing constraints that forced poses to make specific intermolecular interactions with the target. Next, the resulting hIKK-2 complexes were analyzed with the help of LigandScout to determine which complexes exhibited target-inhibitor intermolecular interactions equivalent to those described in prior studies. This knowledge-based analysis enabled us to identify at least one knowledge-based coherent pose (43 in total) for 21 out of the 36 hIKK-2 inhibitors assayed (regardless of their scoring by eHiTS; see their values in the Knowledge-based coherent docking poses column). By analyzing the chemical features used by each pose in its intermolecular interaction with hIKK-2, a common pharmacophore was derived that describes the mechanism of the ligand-target interaction. The Cluster column shows the cluster in which each molecule was classified after running a Schrödinger script that clusters molecules based on Tanimoto similarities between MOLPRINT 2D fingerprints (using the Knime v.2.0.3 module in the Schrödinger software package). The molecules distributed in these clusters are the natural products obtained as hits in our virtual-screening protocol and all known hIKK-2 inhibitors used in the present work [either for validation (see Table S2) or for pharmacophore-generation purposes]. The pIC50 values were obtained from the literature. (PDF) [file pone.0016903.s001.pdf]

Table S1. hIKK-2 inhibitors from different chemical families docked with the hIKK-2 homology model.

2D structure	Molecule name	pIC_{50}	cluster	Knowledge-based coherent docking poses
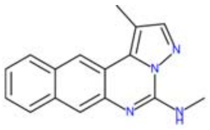	**13**a [1]	7,96	6	3
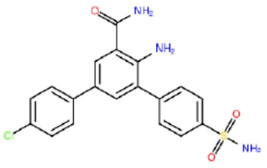	**8h** [2]	7.0	6	0
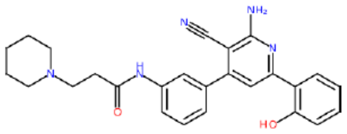	**26** [3]	6,22	6	0
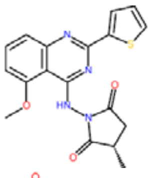	**SCP-839** [4]	8,10	6	0
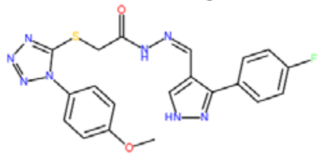	**M8012-3312** [4]	ND	6	0
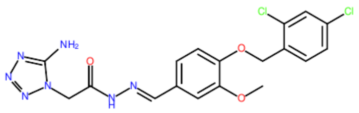	**M8008-5430** [4]	ND	6	0
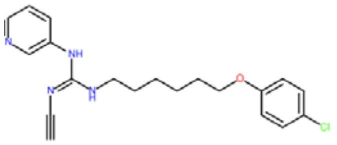	**CHS-828** [4]	8,10	6	0
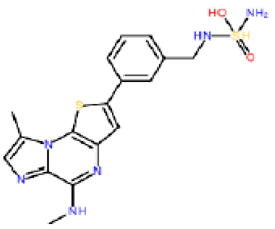	**22d** [5]	8,52	6	1

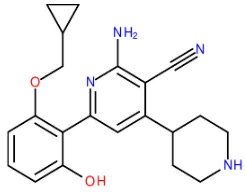	**4j** [6]	8,07	6	1
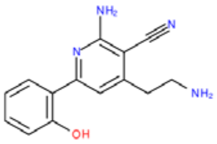	**4a** [7]	7,15	6	4
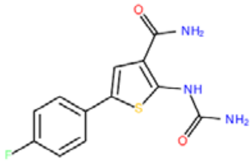	**SKB-TPCA1** [4]	7,74	6	3
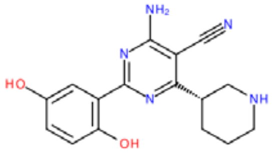	**Pharmacia** [4]	6,50	6	2
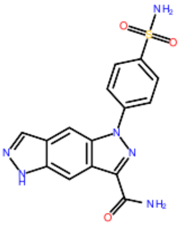	**Pharmacia_02** [4]	6,17	6	4
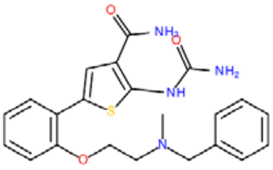	**Astra Zeneca** [4]	7,40	6	1
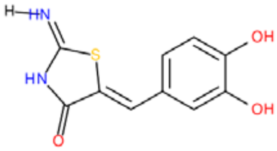	**M4891-3155** [4]	5,75	5	2
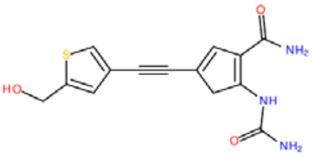	**1b** [8]	6,71	9	2
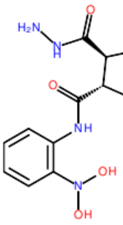	**M2295-0236** [4]	ND	6	0

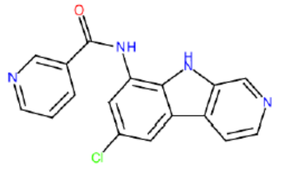	**PS-1145** [9]	6,82	6	0
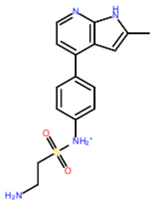	**14** [10]	7,8	6	3
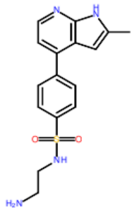	**13** [10]	7,7	6	2
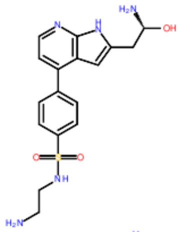	**12** [10]	7,7	6	4
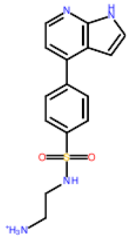	**1** [10]	7,4	6	1
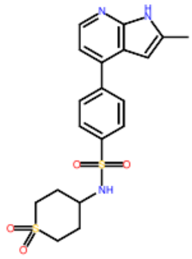	**17** [10]	7,7	6	0
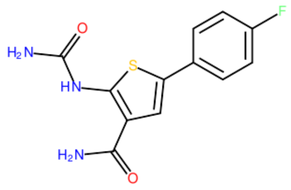	**Ureido** [9]	7,74	6	1

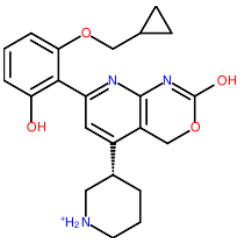	**Compound A** [11]	8,4	6	0
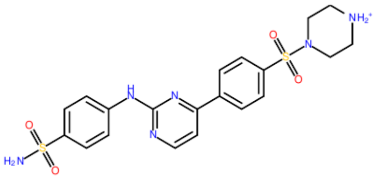	**8u** [12]	7,96	8	0
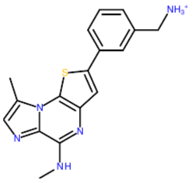	**22a** [5]	8,07	7	1
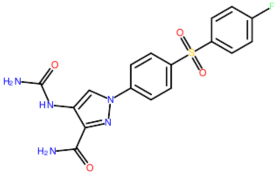	**204** [13]	7,88	8	0
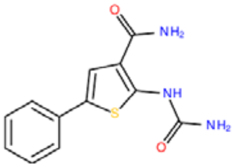	**13** [14]	7,88	8	2
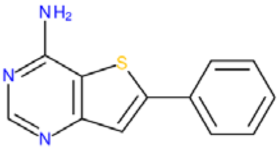	**12** [15]	6,01	8	2
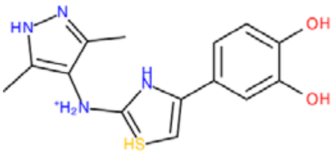	**M7790-1103** [4]	ND	8	0

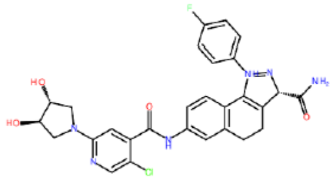	**PF-184** [16]	7,43	6	0
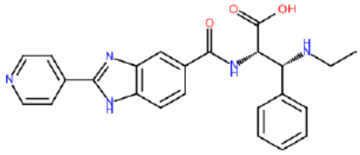	**Aventis** [4]	7,15	6	0
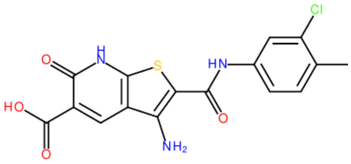	**M4296-0831** [4]	ND	10	1
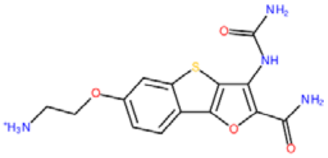	**16k** [17]	7,96	8	2
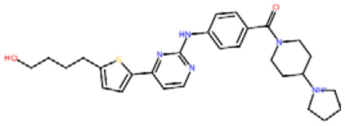	**24** [18]	7,60	9	1

These 36 hIKK-2 inhibitors were docked with our hIKK-2 homology model without imposing constraints that forced poses to make specific intermolecular interactions with the target. Next, the resulting hIKK-2 complexes were analyzed with the help of LigandScout to determine which complexes exhibited target-inhibitor intermolecular interactions equivalent to those described in prior studies. This knowledge-based analysis enabled us to identify at least one knowledge-based coherent pose (43 in total) for 21 out of the 36 hIKK-2 inhibitors assayed (regardless of their scoring by eHiTS; see their values in the *Knowledge-based coherent docking poses* column). By analyzing the chemical features used by each pose in its intermolecular interaction with hIKK-2, a common pharmacophore was derived that describes the mechanism of the ligand-target interaction. The *Cluster* column shows the cluster in which each molecule was classified after running a Schrödinger script that clusters molecules based on Tanimoto similarities between MOLPRINT 2D fingerprints (using the Knime v.2.0.3 module in the Schrödinger software package). The molecules distributed in these clusters are the natural

products obtained as hits in our virtual-screening protocol and all known hIKK-2 inhibitors used in the present work [either for validation (see Table S2) or for pharmacophore-generation purposes]. The pIC_{50} values were obtained from the literature.

REFERENCES

1. Beaulieu F, Ouellet C, Ruediger EH, Belema M, Qiu YP, et al. (2007) Synthesis and biological evaluation of 4-amino derivatives of benzimidazoquinoxaline, benzimidazoquinoline, and benzopyrazoloquinazoline as potent IKK inhibitors. Bioorg Med Chem Lett 17: 1233-1237.

2. Christopher JA, Avitabile BG, Bamborough P, Champigny AC, Cutler GJ, et al. (2007) The discovery of 2-amino-3,5-diarylbenzamide inhibitors of IKK-α and IKK-β kinases. Bioorg Med Chem Lett 17: 3972-3977.

3. Murata T, Shimada M, Sakakibara S, Yoshino T, Kadono H, et al. (2003) Discovery of novel and selective IKK-β serine-threonine protein kinase inhibitors. Part 1. Bioorg Med Chem Lett 13: 913-918.

4. Nagarajan S, Doddareddy M, Choo H, Cho YS, Oh KS, et al. (2009) IKKβ inhibitors identification part I: homology model assisted structure based virtual screening. Bioorg Med Chem 17: 2759-2766.

5. Belema M, Bunker A, Nguyen V, Beaulieu F, Ouellet C, et al. (2007) Synthesis and structure-activity relationship of imidazo(1,2-a)thieno(3,2-e)pyrazines as IKK-β inhibitors. Bioorg Med Chem Lett 17: 4284-4289.

6. Murata T, Shimada M, Sakakibara S, Yoshino T, Masuda T, et al. (2004) Synthesis and structure-activity relationships of novel IKK-β inhibitors. Part 3: Orally active anti-inflammatory agents. Bioorg Med Chem Lett 14: 4019-4022.

7. Murata T, Shimada M, Kadono H, Sakakibara S, Yoshino T, et al. (2004) Synthesis and structure-activity relationships of novel IKK-β inhibitors. Part 2: Improvement of *in vitro* activity. Bioorg Med Chem Lett 14: 4013-4017.

8. Bonafoux D, Bonar S, Christine L, Clare M, Donnelly A, et al. (2005) Inhibition of IKK-2 by 2-[(aminocarbonyl)amino]-5-acetylenyl-3-thiophenecarboxamides. Bioorg Med Chem Lett 15: 2870-2875.

9. Karin M, Yamamoto Y, Wang QM (2004) The IKK NF-κB system: a treasure trove for drug development. Nat Rev Drug Discov 3: 17-26.

10. Liddle J, Bamborough P, Barker MD, Campos S, Cousins RP, et al. (2009) 4-Phenyl-7-azaindoles as potent and selective IKK2 inhibitors. Bioorg Med Chem Lett 19: 2504-2508.

11. Ziegelbauer K, Gantner F, Lukacs N, Berlin A, Fuchikami K, et al. (2005) A selective novel low-molecular-weight inhibitor of IκB kinase-β (IKK-β) prevents pulmonary inflammation and shows broad anti-inflammatory activity. Br J Pharmacol 145: 178-192.

12. Bingham AH, Davenport RJ, Gowers L, Knight RL, Lowe C, et al. (2004) A novel series of potent and selective IKK2 inhibitors. Bioorg Med Chem Lett 14: 409-412.

13. Clare M, Fletcher T, Hamper BC, Hanson G, Heier RF, et al. (2005) Substituted pyrazole urea compounds for the treatment of inflammation. WO/2005/037797.

14. Baxter A, Brough S, Cooper A, Floettmann E, Foster S, et al. (2004) Hit-to-lead studies: the discovery of potent, orally active, thiophenecarboxamide IKK-2 inhibitors. Bioorg Med Chem Lett 14: 2817-2822.

15. Morwick T, Berry A, Brickwood J, Cardozo M, Catron K, et al. (2006) Evolution of the thienopyridine class of inhibitors of IκB kinase-β. Part I: hit-to-lead strategies. J Med Chem 49: 2898-2908.

16. Sommers CD, Thompson JM, Guzova JA, Bonar SL, Rader RK, et al. (2009) Novel tight-binding inhibitory factor-κB kinase (IKK-2) inhibitors demonstrate target-specific anti-inflammatory activities in cellular assays and following oral and local delivery in an *in vivo* model of airway inflammation. J Pharmacol Exp Ther 330: 377-388.

17. Sugiyama H, Yoshida M, Mori K, Kawamoto T, Sogabe S, et al. (2007) Synthesis and structure activity relationship studies of benzothieno[3,2-b]furan derivatives as a novel class of IKKβ inhibitors. Chem Pharm Bull (Tokyo) 55: 613-624.

18. Waelchli R, Bollbuck B, Bruns C, Buhl T, Eder J, et al. (2006) Design and preparation of 2-benzamido-pyrimidines as inhibitors of IKK. Bioorg Med Chem Lett 16: 108-112.
