# Supplementary material for: Identification of Human IKK-2 Inhibitors of Natural Origin (Part I): Modeling of the IKK-2 Kinase Domain, Virtual Screening and Activity Assays
Source: PLoS One. 2011 Feb 24;6(2):e16903. doi: 10.1371/journal.pone.0016903 (PMC3044726; doi:10.1371/journal.pone.0016903)
Supplement: Table S2 — hIKK-2 inhibitors used during the validation of the virtual-screening workflow. These 62 hIKK-2 inhibitors (different from the 36 used during the structure-based pharmacophore generation; see Table S1) were used to test the ability of the virtual-screening workflow to identify hIKK-2 inhibitors in a database of molecules. The Cluster column shows the cluster into which each molecule was classified after running a Schrödinger script that clusters molecules based on Tanimoto similarities between MOLPRINT 2D fingerprints (using the Knime v.2.0.3 module in the Schrödinger software package). The molecules distributed in these clusters are the natural products obtained as hits in our virtual-screening protocol and all known hIKK-2 inhibitors used in the present work (either for validation or for pharmacophore-generation purposes). The pIC50 values were obtained from the literature. (PDF) [file pone.0016903.s002.pdf]

Table S2. hIKK-2 inhibitors used during the validation of the virtual-screening workflow.

2D structure	Molecule name	pIC_{50}	cluster
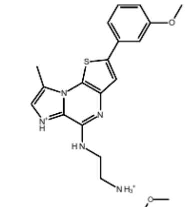	**01** [1]	6.33	4
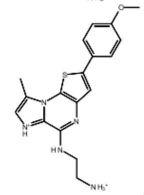	**03** [1]	7.02	4
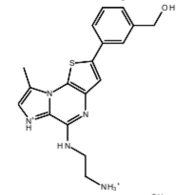	**05** [1]	8.09	4
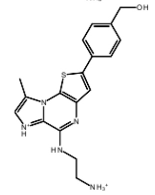	**06** [1]	7.64	4
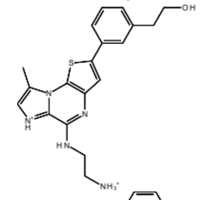	**07** [1]	7.68	4
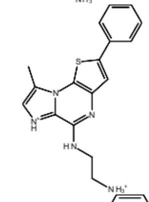	**08** [1]	7.66	4
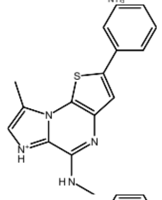	**09** [1]	7.57	4
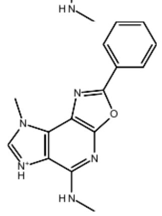	**12** [2]	7.99	4

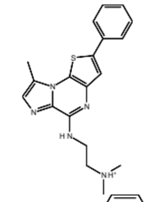	**12** [1]	7.02	4
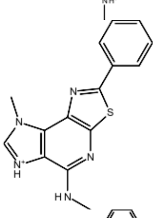	**14** [2]	8.22	4
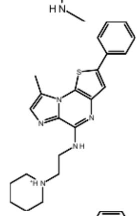	**16** [1]	7.40	4
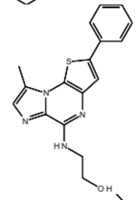	**17** [1]	7.07	4
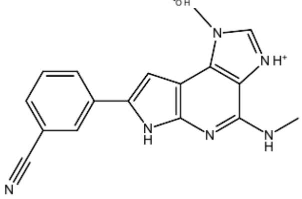	**18** [3]	8.15	4
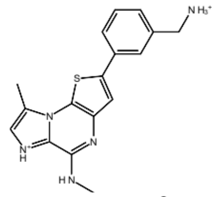	**18** [1]	8.07	4
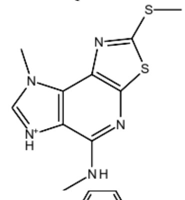	**19** [2]	7.55	4
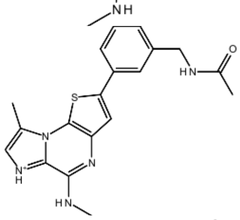	**19** [1]	7.42	4
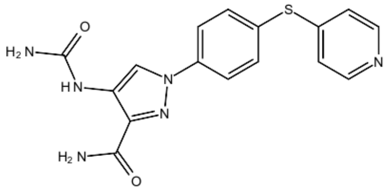	**206** [4]	7.29	8

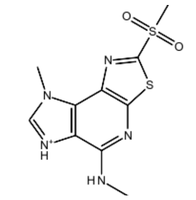	**20** [2]	7.09	4
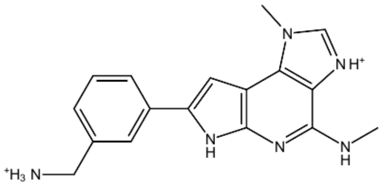	**20** [3]	7.96	4
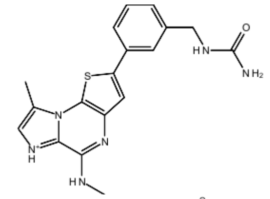	**20** [1]	7.35	4
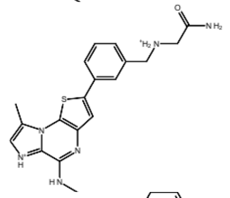	**22** [1]	7.64	4
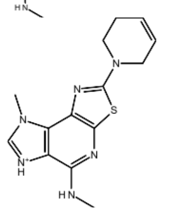	**23d** [2]	7.48	4
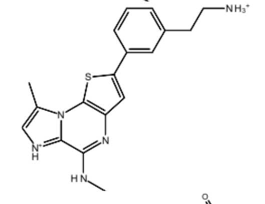	**23** [1]	7.35	4
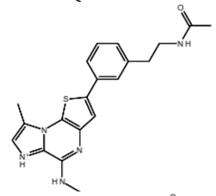	**24** [1]	7.68	4
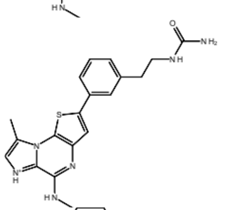	**25** [1]	7.74	4
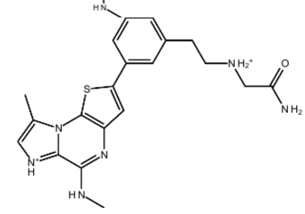	**27** [1]	7.88	4

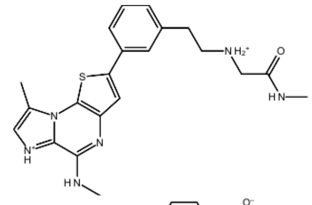	**28** [1]	7.29	4
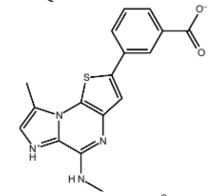	**29** [1]	6.72	4
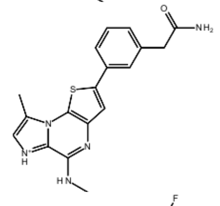	**31** [1]	8.09	4
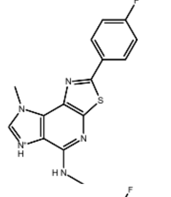	**32a** [2]	7.58	4
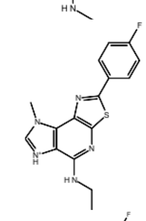	**32b** [2]	7.41	4
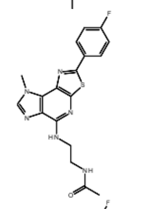	**32d** [2]	7.48	4
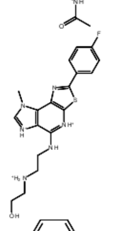	**32e** [2]	7.12	4
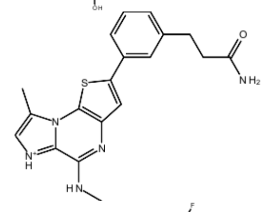	**32** [1]	7.72	4
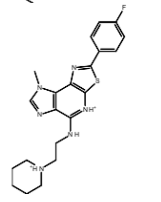	**32f** [2]	7.25	4

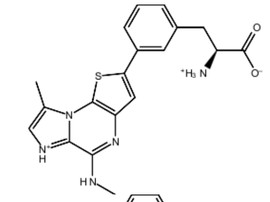	**33** [1]	7.88	4
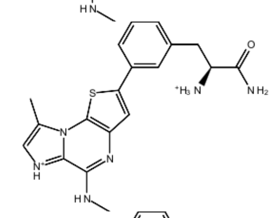	**34** [1]	7.57	4
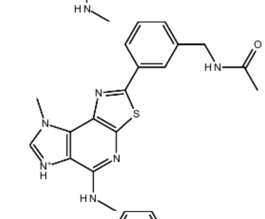	**36a** [2]	7.66	4
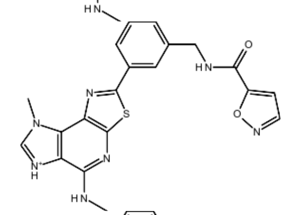	**36c** [2]	7.60	4
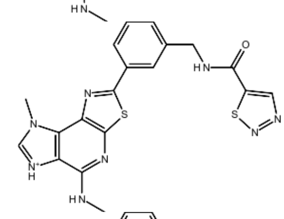	**36d** [2]	7.80	4
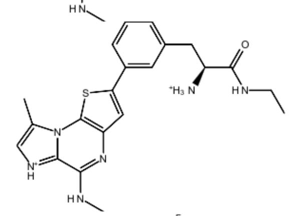	**36** [1]	7.22	4
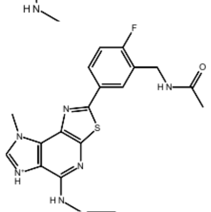	**37a** [2]	7.27	4
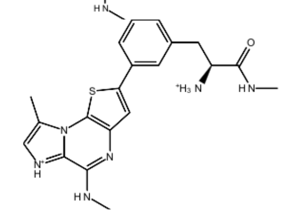	**37** [1]	7.44	4
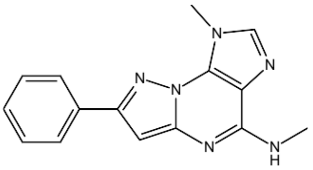	**3b** [2]	7.47	4

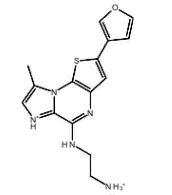	**40** [1]	7.54	4
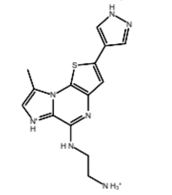	**43** [1]	8	4
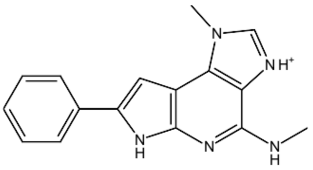	**4a** [3]	7.40	4
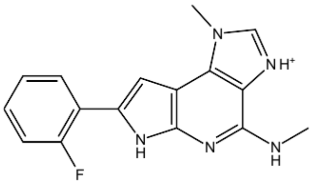	**4b** [3]	7.36	4
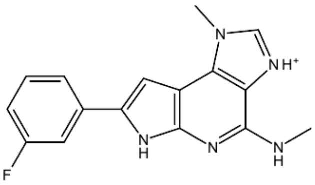	**4c** [3]	8.15	4
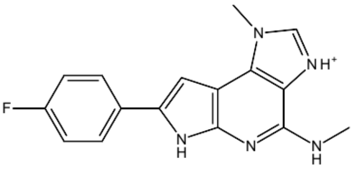	**4d** [3]	7.36	4
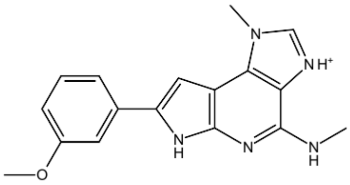	**4e** [3]	8.09	4
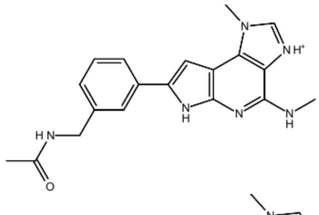	**4f** [3]	7.85	4
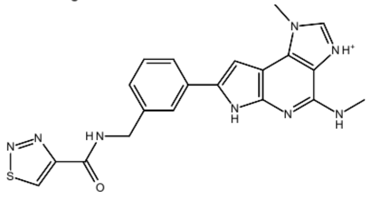	**4h** [3]	7.41	4

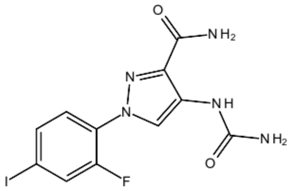	**210** [4]	7.02	8
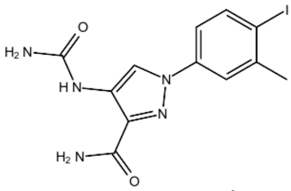	**8** [4]	7.17	8
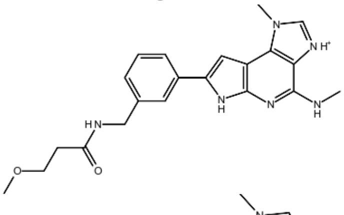	**4k** [3]	7.92	4
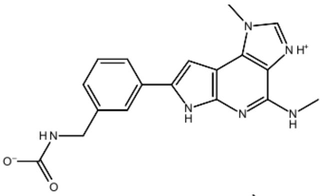	**4g** [3]	7.60	4
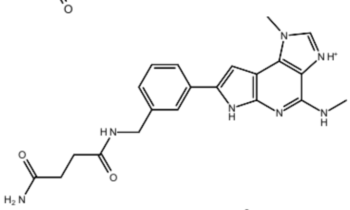	**4j** [3]	7.66	4
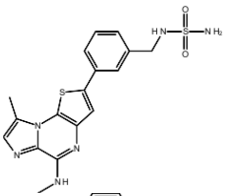	**21** [1]	8.52	4
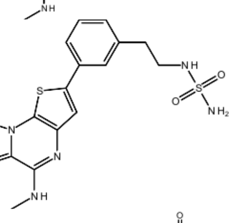	**26** [1]	8.15	4
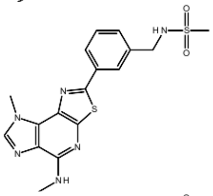	**36b** [2]	8.30	4
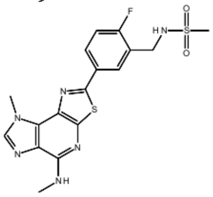	**37b** [2]	7.50	4

These 62 hIKK-2 inhibitors (different from the 36 used during the structure-based pharmacophore generation; see Table S1) were used to test the ability of the virtual-screening workflow to identify hIKK-2 inhibitors in a database of molecules. The *Cluster* column shows the cluster into which each molecule was classified after running a Schrödinger script that clusters molecules based on Tanimoto similarities between MOLPRINT 2D fingerprints (using the Knime v.2.0.3 module in the Schrödinger software package). The molecules distributed in these clusters are the natural products obtained as hits in our virtual-screening protocol and all known hIKK-2 inhibitors used in the present work (either for validation or for pharmacophore-generation purposes). The pIC_{50} values were obtained from the literature.

REFERENCES

1. Wei Long PL, Xinru Li, Yang Xu, Jie Yu, Shitang Ma, Lingling Yu, Zhongmei Zou (2009) QSAR studies on imidazothienopyrazines as IKK-β inhibitors: from 2D to 3D. Journal of Chemiometrics 23: 304-314.

2. Kempson J, Spergel SH, Guo J, Quesnelle C, Gill P, et al. (2009) Novel tricyclic inhibitors of IκB kinase. J Med Chem 52: 1994-2005.

3. Kempson J, Guo J, Das J, Moquin RV, Spergel SH, et al. (2009) Synthesis, initial SAR and biological evaluation of 1,6-dihydroimidazo[4,5-d]pyrrolo[2,3-b]pyridin-4-amine derived inhibitors of IκB kinase. Bioorg Med Chem Lett 19: 2646-2649.

4. Clare M, Fletcher T, Hamper BC, Hanson G, Heier RF, et al. (2005) Substituted pyrazole urea compounds for the treatment of inflammation. WO/2005/037797.
