# Supplementary material for: Identification of Human IKK-2 Inhibitors of Natural Origin (Part I): Modeling of the IKK-2 Kinase Domain, Virtual Screening and Activity Assays
Source: PLoS One. 2011 Feb 24;6(2):e16903. doi: 10.1371/journal.pone.0016903 (PMC3044726; doi:10.1371/journal.pone.0016903)
Supplement: Table S3 — Scaffold-hopping candidates for hIKK-2 inhibition predicted by our study. The ZINC codes for the 246 hit molecules predicted to inhibit hIKK-2 and belonging to clusters consisting exclusively of natural products. For each hit molecule, the best results of the shape and electrostatic-potential comparisons with 43 poses from 21 known hIKK-2 inhibitors (see Table S1) is shown. Thus, the Tanimoto values for the comparison between the electrostatic potentials of the molecules (using an outer dielectric of 80) are shown in the PB columns, whereas the values for the comparison between shapes are shown in the Shape columns. The sum of the PB and Shape values is reported in the Combo columns. Hits from each cluster are sorted according their decreasing combo value. All of these hit molecules are scaffold-hopping candidates for hIKK-2 inhibition because the Tanimoto similarities between their MOLPRINT 2D fingerprints and those from the hIKK-2 inhibitors in Tables S1 and S2 are quite low. ZINC00058225, ZINC01669260 and ZINC16946275 from Cluster 1, ZINC03683886 from Cluster 2, and ZINC03871389 from Cluster 3 were selected to experimentally test the success rate of our predictions using an in vitro assay (in bold in Table S3). The results of this experiment showed that three out of the five molecules (i.e., ZINC01669260 from Cluster 1, ZINC03683886 from Cluster 2 and ZINC03871389 from Cluster 3) inhibited hIKK-2, with IC50 values ranging from 183.8 to 3,325 µM. (PDF) [file pone.0016903.s003.pdf]

Table S3. Scaffold-hopping candidates for hIKK-2 inhibition predicted by our study.

Cluster 1 hits

	PB	Shape	Combo		PB	Shape	Combo
ZINC12410246	0,699	0,528	1,227	ZINC08298038	0,597	0,349	0,946
ZINC04222236	0,727	0,498	1,224	ZINC08298000	0,633	0,308	0,941
ZINC08299593	0,723	0,462	1,185	ZINC04260050	0,609	0,325	0,934
ZINC08300807	0,606	0,560	1,166	ZINC03851724	0,520	0,414	0,933
ZINC08300706	0,697	0,460	1,157	ZINC05415683	0,585	0,345	0,929
ZINC05730981	0,682	0,451	1,134	ZINC08635779	0,579	0,349	0,928
ZINC08299517	0,645	0,489	1,134	ZINC08299591	0,516	0,412	0,928
ZINC04235841	0,626	0,498	1,124	ZINC08299896	0,497	0,429	0,926
ZINC05433728	0,638	0,467	1,105	ZINC08298463	0,580	0,343	0,924
ZINC08298452	0,716	0,382	1,098	ZINC00058208	0,261	0,661	0,923
ZINC08635299	0,677	0,414	1,091	ZINC08299131	0,538	0,378	0,917
ZINC08299522	0,632	0,458	1,091	ZINC08297955	0,444	0,472	0,917
ZINC12604815	0,542	0,547	1,089	ZINC08300143	0,629	0,287	0,916
ZINC08623382	0,618	0,457	1,075	ZINC03841142	0,575	0,341	0,916
ZINC08298456	0,627	0,441	1,068	ZINC00195221	0,491	0,424	0,915
ZINC05433715	0,601	0,466	1,068	ZINC02023740	0,175	0,739	0,914
ZINC03841282	0,686	0,380	1,066	ZINC01204686	0,644	0,267	0,911
ZINC04028468	0,633	0,429	1,062	ZINC08298449	0,588	0,321	0,910
ZINC08296915	0,695	0,361	1,056	ZINC04082733	0,528	0,381	0,909
ZINC04222674	0,515	0,540	1,055	**ZINC01669260**	**0,512**	**0,397**	**0,909**
ZINC08299611	0,629	0,425	1,054	ZINC04222254	0,483	0,426	0,909
ZINC04235840	0,629	0,420	1,049	ZINC08299700	0,595	0,313	0,907
ZINC08300121	0,641	0,407	1,048	ZINC03841141	0,595	0,307	0,902
ZINC03841201	0,608	0,439	1,048	ZINC08298553	0,563	0,337	0,901
ZINC08297952	0,648	0,394	1,043	ZINC08296886	0,327	0,573	0,900
ZINC05433681	0,637	0,403	1,040	ZINC08298004	0,441	0,458	0,899
ZINC04235837	0,603	0,424	1,027	ZINC05397696	0,467	0,432	0,898
ZINC08299759	0,530	0,490	1,020	ZINC12604970	0,593	0,303	0,896
ZINC04260104	0,648	0,370	1,018	ZINC00485710	0,468	0,427	0,896
ZINC08296937	0,696	0,319	1,015	ZINC04235971	0,415	0,481	0,896
ZINC08298035	0,662	0,346	1,009	ZINC04236053	0,519	0,373	0,892
ZINC00460033	0,504	0,500	1,004	ZINC08296889	0,415	0,476	0,891
ZINC04235839	0,618	0,379	0,997	**ZINC00058225**	**0,231**	**0,660**	**0,891**
ZINC04222682	0,591	0,403	0,993	ZINC05434223	0,476	0,412	0,888
ZINC08254138	0,661	0,331	0,992	ZINC08254330	0,641	0,243	0,883
ZINC00035451	0,679	0,313	0,991	ZINC08299765	0,364	0,518	0,882
ZINC05396170	0,589	0,402	0,991	ZINC08298536	0,519	0,362	0,881
ZINC08254141	0,678	0,312	0,990	ZINC08299629	0,543	0,335	0,878
ZINC03838691	0,598	0,390	0,989	ZINC05433729	0,385	0,492	0,877
ZINC00485569	0,604	0,381	0,986	ZINC05279694	0,487	0,384	0,871
ZINC08299569	0,589	0,396	0,985	ZINC04235956	0,581	0,288	0,869
ZINC00058275	0,329	0,657	0,985	ZINC08296677	0,487	0,382	0,869
ZINC08296946	0,689	0,295	0,984	ZINC04236052	0,495	0,373	0,868
ZINC04236050	0,505	0,479	0,984	ZINC05433716	0,381	0,487	0,868
ZINC04222681	0,591	0,391	0,982	ZINC04236049	0,501	0,365	0,866
ZINC00035452	0,592	0,388	0,979	ZINC08300122	0,496	0,370	0,866
ZINC08300479	0,535	0,443	0,978	ZINC01601221	0,204	0,662	0,866
ZINC08299635	0,641	0,336	0,977	ZINC08298097	0,638	0,227	0,865
ZINC08300733	0,613	0,363	0,976	ZINC04235961	0,592	0,272	0,864
ZINC12604292	0,547	0,424	0,971	ZINC08254273	0,509	0,352	0,861
ZINC03838674	0,578	0,390	0,968	**ZINC16946275**	**0,504**	**0,357**	**0,861**
ZINC08254375	0,562	0,405	0,966	ZINC04236055	0,517	0,343	0,860
ZINC08298030	0,668	0,296	0,963	ZINC04236051	0,509	0,350	0,859
ZINC00485709	0,396	0,565	0,961	ZINC04235957	0,582	0,274	0,856
ZINC05433719	0,494	0,462	0,957	ZINC08298002	0,555	0,301	0,856
ZINC04260127	0,594	0,360	0,954	ZINC12604752	0,439	0,416	0,855
ZINC08298652	0,570	0,382	0,953	ZINC03841193	0,256	0,598	0,854
ZINC08254451	0,664	0,287	0,951	ZINC04236054	0,503	0,350	0,853
ZINC04260095	0,624	0,327	0,951	ZINC00048191	0,498	0,355	0,853
ZINC08298659	0,619	0,328	0,947	ZINC08299638	0,616	0,235	0,851

Cluster 2 hits

	PB	Shape	Combo		PB	Shape	Combo
ZINC00485744	0,723	0,480	1,203	ZINC05224098	0,458	0,464	0,922
ZINC03838566	0,704	0,475	1,179	ZINC02151124	0,485	0,426	0,911
ZINC00346993	0,585	0,586	1,170	ZINC12653223	0,305	0,606	0,910
ZINC00094845	0,580	0,587	1,167	ZINC03842171	0,424	0,474	0,898
ZINC00485534	0,651	0,508	1,159	ZINC09231861	0,627	0,264	0,891
ZINC13424993	0,544	0,613	1,157	ZINC00040813	0,228	0,662	0,890
ZINC11616540	0,709	0,433	1,141	ZINC05224529	0,511	0,376	0,888
ZINC00227258	0,660	0,477	1,137	ZINC00517451	0,381	0,504	0,885
ZINC00485860	0,623	0,510	1,132	ZINC01317724	0,224	0,660	0,884
ZINC08762138	0,497	0,623	1,120	ZINC12604995	0,600	0,284	0,883
ZINC00036355	0,626	0,487	1,113	ZINC03786448	0,250	0,633	0,883
ZINC12657772	0,505	0,607	1,112	ZINC05224397	0,553	0,329	0,881
ZINC02124459	0,562	0,541	1,104	ZINC00039884	0,342	0,536	0,878
ZINC04081954	0,524	0,564	1,088	ZINC12604997	0,615	0,261	0,876
ZINC08738350	0,566	0,521	1,087	ZINC11616541	0,598	0,276	0,875
ZINC08762259	0,510	0,573	1,082	ZINC02090323	0,423	0,452	0,875
ZINC00388584	0,665	0,415	1,080	ZINC03852423	0,444	0,430	0,874
ZINC00130101	0,614	0,464	1,078	ZINC02027203	0,300	0,573	0,874
ZINC00180655	0,603	0,471	1,074	ZINC03898167	0,461	0,410	0,871
ZINC01646905	0,584	0,490	1,074	ZINC13424994	0,406	0,463	0,868
ZINC01102508	0,606	0,459	1,065	ZINC13306086	0,492	0,368	0,860
ZINC04027790	0,524	0,536	1,060	ZINC01081585	0,400	0,460	0,860
ZINC12318138	0,590	0,462	1,052	ZINC13306051	0,488	0,371	0,859
ZINC01854651	0,572	0,475	1,048	ZINC03984208	0,382	0,476	0,858
ZINC00044054	0,541	0,505	1,046	ZINC01665127	0,337	0,519	0,856
ZINC00517161	0,582	0,461	1,044	ZINC00369448	0,445	0,410	0,855
ZINC01321570	0,563	0,478	1,041	ZINC05224220	0,404	0,449	0,853
ZINC04104779	0,547	0,489	1,036	ZINC01556769	0,245	0,606	0,851
ZINC01950801	0,631	0,401	1,031				
ZINC00485558	0,717	0,313	1,029				
ZINC13425030	0,590	0,436	1,027				
ZINC12318136	0,585	0,442	1,027				
ZINC12656099	0,531	0,493	1,024				
ZINC02124460	0,583	0,438	1,021				
ZINC04032263	0,531	0,488	1,019				
ZINC00485559	0,505	0,504	1,009				
ZINC13425093	0,670	0,336	1,006				
ZINC01842501	0,545	0,457	1,002				
ZINC03978029	0,527	0,473	0,999				
ZINC02372275	0,631	0,366	0,996				
ZINC13306038	0,456	0,540	0,996				
ZINC12655829	0,518	0,476	0,994				
ZINC05224095	0,446	0,543	0,989				
ZINC13425031	0,593	0,392	0,985				
ZINC12658812	0,516	0,462	0,979				
**ZINC03683886**	**0,559**	**0,419**	**0,977**				
ZINC00517162	0,533	0,445	0,977				
ZINC01321569	0,603	0,362	0,965				
ZINC08552275	0,566	0,398	0,964				
ZINC04027791	0,414	0,544	0,958				
ZINC01679223	0,293	0,661	0,954				
ZINC12657776	0,565	0,387	0,952				
ZINC12428234	0,655	0,291	0,946				
ZINC08762235	0,471	0,471	0,942				
ZINC03838563	0,569	0,369	0,939				
ZINC08299557	0,429	0,507	0,937				
ZINC12661595	0,522	0,414	0,936				
ZINC03898166	0,475	0,457	0,932				
ZINC12660883	0,664	0,263	0,928				
ZINC08740325	0,499	0,428	0,927				

Cluster 3 hits

	PB	Shape	Combo
ZINC00071662	0,536	0,665	1,201
ZINC00138773	0,742	0,438	1,180
ZINC00525792	0,727	0,435	1,162
ZINC00126518	0,627	0,444	1,071
**ZINC03871389**	**0,408**	**0,630**	**1,038**
ZINC13404337	0,341	0,659	0,999
ZINC00971251	0,485	0,506	0,992
ZINC06624596	0,341	0,636	0,976
ZINC00405375	0,363	0,603	0,966
ZINC05396117	0,542	0,418	0,960
ZINC00126516	0,253	0,699	0,952
ZINC13402114	0,610	0,339	0,949
ZINC00262010	0,388	0,555	0,943
ZINC00526037	0,456	0,483	0,939
ZINC00518532	0,397	0,537	0,934
ZINC00519161	0,339	0,593	0,932
ZINC04280948	0,342	0,581	0,923
ZINC13404329	0,282	0,638	0,920
ZINC13110434	0,322	0,588	0,910
ZINC02136236	0,621	0,288	0,909
ZINC03842020	0,556	0,346	0,901
ZINC04261011	0,481	0,420	0,901
ZINC00519153	0,270	0,628	0,898
ZINC00518538	0,193	0,705	0,898
ZINC12341518	0,336	0,561	0,896
ZINC00517656	0,338	0,556	0,894
ZINC00525794	0,591	0,302	0,893
ZINC00189849	0,360	0,529	0,890
ZINC03842034	0,515	0,369	0,884
ZINC03844523	0,284	0,599	0,883
ZINC13402109	0,425	0,449	0,875
ZINC04504120	0,448	0,418	0,866
ZINC04260974	0,553	0,310	0,863
ZINC00178183	0,239	0,622	0,861
ZINC03850520	0,518	0,342	0,860
ZINC00518609	0,227	0,633	0,860
ZINC00296237	0,429	0,424	0,853
ZINC00042159	0,260	0,591	0,851

The ZINC codes for the 246 hit molecules predicted to inhibit hIKK-2 and belonging to clusters consisting exclusively of natural products. For each hit molecule, the best results of the shape and electrostatic-potential comparisons with 43 poses from 21 known hIKK-2 inhibitors (see Table S1) is shown. Thus, the Tanimoto values for the comparison between the electrostatic potentials of the molecules (using an outer dielectric of 80) are shown in the *PB* columns, whereas the values for the comparison between shapes are shown in the *Shape* columns. The sum of the PB and Shape values is reported in the *Combo* columns. Hits from each cluster are sorted according their decreasing combo value. All of these hit molecules are scaffold-hopping candidates for hIKK-2 inhibition because the Tanimoto similarities between their MOLPRINT 2D fingerprints and those from the hIKK-2 inhibitors in Tables S1 and S2 are quite low. ZINC00058225, ZINC01669260 and ZINC16946275 from Cluster 1, ZINC03683886 from Cluster 2, and ZINC03871389 from Cluster 3 were selected to experimentally test the success rate of our predictions using an *in vitro* assay (in bold in Table S3). The results of this experiment showed that three out of the five molecules (*i.e.*, ZINC01669260 from Cluster 1, ZINC03683886 from Cluster 2 and ZINC03871389 from Cluster 3) inhibited hIKK-2, with IC_{50} values ranging from 183.8 to 3,325 µM.
